# Dietary excess regulates absorption and surface of gut epithelium through intestinal PPARα

**DOI:** 10.1038/s41467-021-27133-7

**Published:** 2021-12-02

**Authors:** Ozren Stojanović, Jordi Altirriba, Dorothée Rigo, Martina Spiljar, Emilien Evrard, Benedek Roska, Salvatore Fabbiano, Nicola Zamboni, Pierre Maechler, Françoise Rohner-Jeanrenaud, Mirko Trajkovski

**Affiliations:** 1 Department of Cell Physiology and Metabolism, Centre Medical Universitaire (CMU), Faculty of Medicine, University of Geneva, Geneva, Switzerland; 2 Diabetes Centre, Faculty of Medicine, University of Geneva, 1211 Geneva, Switzerland; 3 Institute for Molecular Systems Biology, Swiss Federal Institute of Technology (ETH) Zurich, 8093 Zurich, Switzerland; 4 Present Address: Department of Physiology, Anatomy and Genetics, University of Oxford, Oxford, OX1 3PT UK

**Keywords:** Small intestine, Obesity

## Abstract

Intestinal surface changes in size and function, but what propels these alterations and what are their metabolic consequences is unknown. Here we report that the food amount is a positive determinant of the gut surface area contributing to an increased absorptive function, reversible by reducing daily food. While several upregulated intestinal energetic pathways are dispensable, the intestinal PPARα is instead necessary for the genetic and environment overeating–induced increase of the gut absorptive capacity. In presence of dietary lipids, intestinal PPARα knock-out or its pharmacological antagonism suppress intestinal crypt expansion and shorten villi in mice and in human intestinal biopsies, diminishing the postprandial triglyceride transport and nutrient uptake. Intestinal PPARα ablation limits systemic lipid absorption and restricts lipid droplet expansion and PLIN2 levels, critical for droplet formation. This improves the lipid metabolism, and reduces body adiposity and liver steatosis, suggesting an alternative target for treating obesity.

## Abstract (web summary)

Intestinal surface changes in size and function, but what propels these alterations is unknown. Here, the authors show that food excess increases the gut absorptive capacity, and that in presence of dietary lipids, intestinal PPARα is indispensable for the adaptive increase in villi length and function.

## Introduction

Obesity is caused by an energetic disbalance following chronic excess of caloric intake over energy expenditure. Which of the two is more decisive in the modern setting has long been debated^1,2^. Almost all caloric uptake takes place in intestine. However, understanding the relationship between overeating, gut absorption, and obesity is in its infancy. We and others have shown that environmental^3^ or nutritional^4^ stimuli induce changes in gut morphology^5^, which may alter efficiency of caloric uptake^3,6^, contributing to energy homeostasis. While the data on the correlation between the intestinal, villi and microvilli length and the body mass index (BMI) in humans^7–10^ is scarce and contradicting, intestinal hyperplasia is a frequent feature of diabetic hyperglycaemia^9,11^.

Maintenance and expansion of the gut epithelium depends on the activity of Lgr5^+^ intestinal stem cells (ISCs)^12^, which give rise to Paneth cells and rapidly dividing transitory progenitors in intestinal crypts. Along the villi, these cells differentiate into enterocytes, enteroendocrine cells and goblet cells that eventually undergo apoptosis and shedding near the top^13^. Caloric restriction, through Paneth cell mediation, reduces progenitor differentiation and villi length^14^, whereas obese *db/db* mice have longer villi and larger intestines^15^. High-fat diet (HFD) supports division of intestinal stem cells^16^ and tumorigenicity^17^; however, mice fed HFD end up having shorter intestines and villi^4,16^. In contrast, energy-consuming cold exposure shifts intestinal homeostasis towards longer guts and villi^3^, in concert with increased caloric uptake in conditions of increased metabolic demand.

Another metabolic consideration unique to the intestine is that it must distribute nutrients for the rest of the organism while maintaining high metabolic rate for its own needs. As a result, the intestine is a metabolically diversified organ with a complex interplay between processes in the crypt and gut plasticity. ISCs metabolically rely on carbon substrates from Paneth cells^18^ and their own oxidative metabolism^19^. In enterocytes, glutamate is the preferred substrate for oxidation, and is oxidised in both fed (from lumen) and fasted state (from deaminated glutamine), while sugars and fatty acids are released for use elsewhere^20,21^. The entire epithelium is turned over in 3–5 days, which is among the fastest regenerative processes in the body—300 million intestinal epithelial cells need to be generated daily in human to compensate for the losses^13^. Not surprisingly, intestinal homeostasis is energetically costly, and thus likely dependent on energy availability that would in turn control the regenerative processes of the crypt machinery. Changes in intestinal metabolism can affect caloric harvest efficacy^22^, and exert systemic effects on whole body physiology, for example through intestinal gluconeogenesis on hepatic glucose production^23^. Intestinal glycolysis and gluconeogenesis are strongly activated by gastric bypass surgery^24,25^, contributing to its post-resection growth and function. Understanding the metabolic fuelling that enables increase in the intestinal absorptive function holds enormous therapeutic potential. However, the mechanisms and dietary triggers that promote these processes remain elusive. It is also not established how changes in enterocyte metabolism affect nutrient absorption efficiency, both overall and of specific macronutrient.

In this work, we investigate the energy metabolism of the adult gut plasticity triggered by nutritional changes and address the physiological consequences of intestinal metabolic perturbations. We show that intestinal absorptive surface and function are regulated by the amount of consumed food, causing an adaptive increase in the intestinal absorptive capacity in conditions of increased food availability. Using multiple intestine-specific genetic mouse knockouts and human intestinal biopsies, we systematically addressed what metabolic pathways are decisive in controlling the gut plasticity and function. Our work demonstrates that in presence of dietary lipids, PPARα-dependent transcription programs are necessary for enlargement of the intestinal surface through increasing villi length, and for both overall nutrient and triglyceride uptake by enterocytes. Unexpectedly, while PPARα promotes catabolism and fatty acid oxidation in various organs^26,27^, we found that intestinal PPARα deletion or its pharmacological antagonism leads to concomitant reduction of lipid droplet (LD) amount and size, decreased fatty acid transport, and depletion of perilipin 2 (PLIN2), a critical regulator of LD formation. Our work thus proposes that in presence of dietary lipids, the intestinal LD formation and trafficking regulated by PPARα are important rate-limiting steps in the systemic lipid metabolism, and in the food amount-driven intestinal surface enlargement.

## Results

### The amount of ingested food dictates the intestinal absorptive surface and capacity

**Fig. 1:**
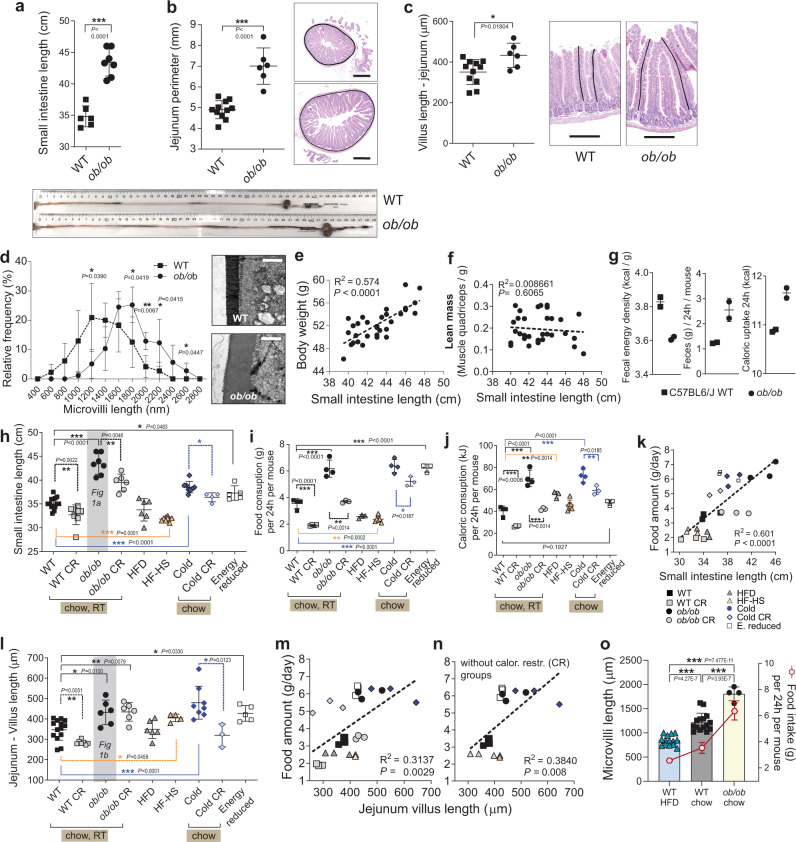
Food amount regulates the intestinal absorptive surface at multiple levels. **a**–**d** Small intestinal length (*n* = 6 WT, *n* = 7 *ob/ob*), repeated in five independent experiments with similar results, and the representative gut images (**a**), perimeter of jejuna (average from *n* = 11 WT, *n* = 6 *ob/ob*) and representative haematoxylin and eosin (H&E) images with perimeter tracing, scale bar: 500 μm (**b**), average lengths of villi in jejuna (average from *n* = 11 WT, *n* = 6 *ob/ob*) with the examples of a villus length measurement on H&E staining, scale bar: 200 μm (**c**), distribution of microvilli lengths (from WT *n* = 5 mice, *ob/ob* *n* = 4 mice) measured along the middle part of villi in proximal jejuna, with the representative electron micrographs, scale bar = 2000 nm (**d**) of *ob/ob* and WT C57BL6/J male mice, 14 week-old. **e**, **f** Linear regression of body weights and small intestinal lengths (**e**) and their lean mass (represented by the weight of the quadriceps muscle) vs. intestinal length (**f**) of *ob/ob* mice pooled from multiple experiments, age 12–14 weeks, *n* = 33 male mice. **g** Caloric density of faecal samples (left), daily faecal output (centre), and total caloric uptake (right, calories from food – excreted calories) in 24 h (right) from WT and *ob/ob* mice, two samples per condition, where each sample is a 24 h faeces collected from a single cage with three mice. **h**–**n** Small intestine length (**h**), daily food intake (**i**), daily caloric consumption (**j**), linear regression of average food intake and intestinal length (**k**), average villi length in jejuna (**l**), linear regression of villi length in jejuna and food intake with (**m**) or without (**n**) calorically restricted groups of male mice under different dietary regimes, 14 week-old, C57BL/6 J background, SPF facility. Abbreviation of the treatments: CR caloric restriction (60% of ad libitum) for six weeks (WT, *ob/ob* groups) or 10 days (cold), HFD high-fat diet, HF-HS high-fat high-sucrose diet, RT room temperature (23 °C), Cold 6 °C for 30 days, Energy reduced ad libitum feeding on diet with the low caloric density for 30 days. For i-n the WT group is pooled from all the respective experiments (**o**), Mean lengths of microvilli from middle part of jejuna of WT mice fed HFD (*n* = 14 mice) or standard chow (*n* = 15), and of *ob/ob* mice (*n* = 4) and their average food intake. Shaded data in (**h**) and (**l**) are repeated data from (**a**) and (**b**). For (**h**), *n* = 14 (WT), eight (WT CR), seven (*ob/ob*), six (*ob/ob* CR), six (HFD), eight (HFHS), 8 (Cold), four (Cold CR) mice. For l, *n* = 14 (WT), seven (WT CR), six (*ob/ob, ob/ob*, HFD), four (HFHS), eight (Cold), three (Cold CR), five (ER) mice. For (**i**), (**j**), *n* = 4 cages (WT, WT CR, ob/ob, Cold), *n* = 3 (ob/ob CR, HFD, Cold CR, Energy reduced), *n* = 6 cages (HF-HS). For (**k**), (**m**), (**n**), *n* = 3 per group, where each dot represents average intestinal or villus length and food intake in a single cage. All data represent mean ± S.D. Statistical tests: for (**h**), (**i**), (**j**), l one-way ANOVA, Dunnet’s post-hoc correction for multiple comparisons (all groups compared to WT), the alpha 0.05 (solid lines), or two-sided *t*-test (dashed lines); for (**k**), (**m**), (**n**) linear regression, confidence level 95%; for all other panels unpaired two-sided *t*-test, confidence level 95%. *P ≤ 0.05, **P < 0.01, ***P < 0.001. Source data are provided as a Source Data file.

We first examined intestinal size in genetically obese mice. Both *ob/ob* and *db/db* mice spontaneously overeat throughout their lifetime due to missing satiety signal, conferred by the lack of leptin or its receptor, respectively^28^. Compared to WT mice, *ob/ob* mice had longer and heavier small intestines (Fig. 1a, Supplementary Fig.1a), similar to the increase seen in *db/db* mice (Supplementary Fig.1b). The perimeter of the jejunum was larger (Fig. 1b), and villi and microvilli longer in *ob/ob* jejuna (Fig. 1c, d), thereby vastly increasing the absorptive surface at multiple levels. In *ob/ob* mice, where increased body weight is primarily due to increased fat accumulation, the length of the small intestines correlated with the body weight (Fig. 1e), while being independent of the lean (muscle) mass (Fig. 1f). Analysis of faeces by bomb calorimetry revealed increased caloric extraction from ingested food in *ob/ob* mice (Fig. 1g). Accordingly, increase in absorptive surface of the genetically-induced overeating obese mice correlated with increased body weight and caloric uptake from the intestine.

To determine if intestinal size correlates with food intake, we compared intestinal sizes (Fig. 1h) in *ob/ob* and in several groups of C57BL/6 J (WT) male mice (Charles River France) at 14 weeks of age, which differed in the amount of eaten food (Fig. 1i) due to exposure to a different temperature, food type or caloric restriction. *Ob/ob* mice ate 50% more standard chow food than WT. However, when the amount of chow given to *ob/ob* was restricted to the same amount as eaten by WT during six weeks (*ob/ob* CR), the small intestine of *ob/ob* decreased in length and width (Fig. 1h, Supplementary Fig.1c). Similarly, WT mice calorically restricted to 60% of ad libitum standard chow, decreased their intestinal length (Fig. 1h). Intestinal length increased in mice where overeating was induced by a 30-day 6 °C exposure. This increment was attenuated when food amount was restricted from *ad libitum* to the minimal necessary levels for 6 °C exposure (Fig. 1h, j). To further address if the amount of eaten food per se, rather than obesity drives the intestinal enlargement, we assessed the gut surface following multiple feeding regiments with food of different caloric densities and food intakes. Mice eat smaller quantities of high-fat diet than of standard chow due to higher energy density of HFD, but that still translates to more calories taken in (Fig. 1i, j). Depending on the exact type of high-fat diet, intestinal length was unchanged or shortened compared to normal chow-fed mice. The calorically most dense high-fat, high-sucrose diet (HF-HS), and fibre-poor, butter-derived HFD shortened the gut (Fig. 1i, Supplementary Fig.1d, Supplementary Table 2). In an interesting contrast to HFD, the energetically depleted food (lower caloric density per gram) demanded overeating and led to gut extension (Fig. 1h, i). A comparison of these conditions revealed a correlation between average intestinal length and the weight of consumed food (Fig. 1k). We found a positive relationship between food intake and average villus length in jejunum (Fig. 1l–n). Food intake and microvilli length of the jejunal brush border, strongly matched in WT HFD, WT chow, and *ob/ob* chow mice. (Fig. 1o). Consistent with the recent report^29^, HF-HS mice had slightly increased villi length likely due to the fructose present in this diet. The correlation between food amount and intestinal plasticity was further tested against variations that could arise from different food mixtures and mice suppliers. Three chow mixtures from different suppliers caused similar food intake and intestinal length (Supplementary Fig.1e, g), though interestingly, a protein-rich, soya and fish-derived diet had increased starting villi length compared to other two vegetable-based foods (Supplementary Fig.1f). C57BL/6 J from Janvier had longer intestines on chow diet compared to mice obtained through Charles River, and HFD produced stronger surface decrease in these mice (Supplementary Fig.1h, i). This suggests that factors such are macronutrient composition or microbiota can influence starting intestinal surface, which is then strongly modified by the food intake. Taken together, these data show that while keeping constant genetic background, age, sex, SPF housing, the intestinal absorptive area positively correlates with the amount of eaten food in respective mice, independently of the caloric intake from that food.

### Multiple energetic pathways are upregulated, but dispensable for the increased intestinal surface and function in vivo

**Fig. 2:**
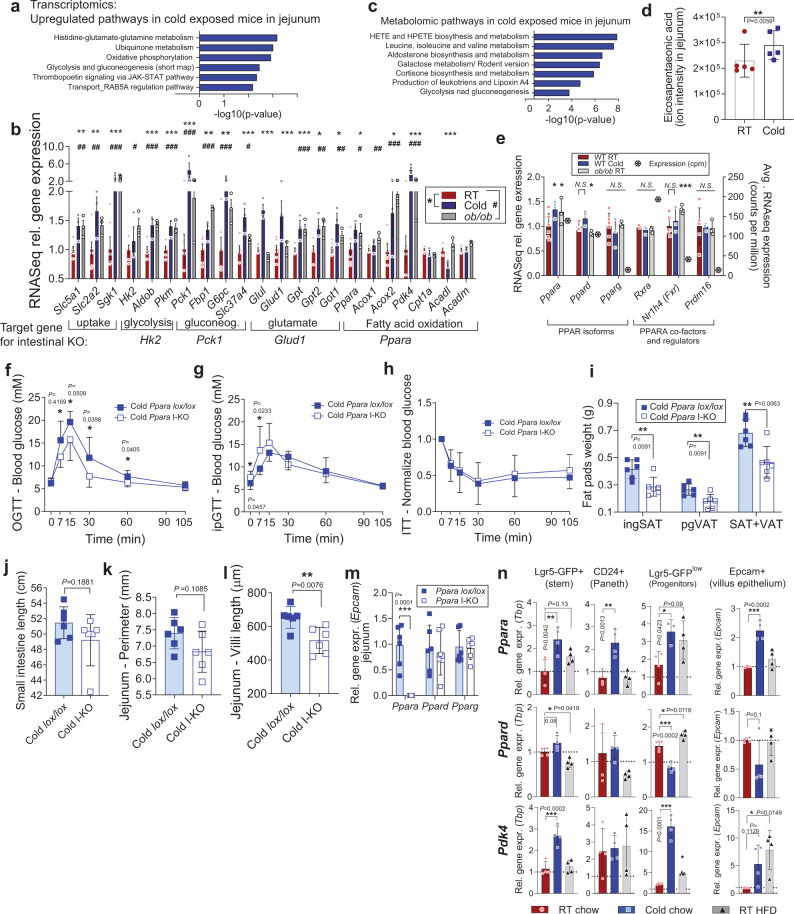
Energy harvesting pathways are upregulated in enlarged intestines of overeating mice, among which *Ppara* is necessary for villi elongation. **a** RNASeq pathway analysis by MetaCore upregulated pathways in jejuna of 30-day cold-exposed WT mice (Cold) compared to room temperature (RT) mice, *n* = 3 sample per group, each sample is a pool of two biological replicates. **b** Relative RNASeq expression of metabolic genes in RT and Cold mice as in a, and in male *ob/ob* mice, *n* = 3 per group, where each sample is a pool of two proximal jejuna. RT *n* = 3 vs. Cold *n* = 3 (*), and WT *n* = 3 vs. ob/ob *n* = 3 (#) comparisons are from separate experiments, Counts normalized and RT and WT pooled together in the figure. **c** Enriched metabolomic pathways in Cold mice jejuna (the whole tissue lysate), for metabolite fold-change *P* < 0.05, as implemented in MetaCore, *n* = 5 mice per group. **d** Relative concentration of eicosapentaenoic acid in jejuna of RT and Cold mice, *n* = 5 per group (*P* = 0.00594, z-scores of the replicates with a z-test, uncorrected for multiple comparison). **e** RNASeq expression of PPAR-related genes in RT, Cold and *ob/ob* mice as in (**b**), together with the average count per million in the RT mice, * and # as in (**b**). **f**–**m** oral glucose tolerance test (**f**), intraperitoneal GTT (**g**), insulin tolerance test (**h**), white adipose tissue pads (ingSAT inguinal subcutaneous adipose tissue, pgVAT perigonadal visceral) (**i**), small intestinal length (**j**), average perimeter of jejunum (**k**) and average villi length in jejunum (**j**) of 30-days cold-exposed *Ppara lox/lox* and *Ppara* I-KO male mice, 16 week-old, *n* = 6 mice per group. **m** Gene expression by qPCR of Ppar isoforms in jejunum tissue of mice from (**f**–**m**). **n** Gene expression by qPCR of *Ppara*, *Ppard*, and PPAR target *Pdk4* in FACS-sorted jejunal cells from Lgr5-EGFP mice exposed two weeks to cold (6 °C) or HFD on RT, from *n* = 4 mice per group. Levels are normalized to *Tbp*, and to room temperature stem cell values (for crypt cell types). All data represent mean ± S.D. *P ≤ 0.05, **P < 0.01, ***P < 0.001 of unpaired two-sided *t*-test, confidence level 95%, except for (**b**) and (**e**), were *P* values for RNASeq data were calculated using general linear model with negative binomial distribution, no correction for multiple comparison, for (**d**), z-scores, and for (**m**), one-way ANOVA, Dunnet’s post-hoc correction for multiple comparison, alpha 0.05. Source data are provided as a Source Data file, including exact *P* values for panels (**b**) and (**e**).

Next, we systematically analysed the upregulation of metabolic pathways in elongated guts. Pathway enrichment analyses of transcriptomes from jejuna of cold-exposed mice revealed that energy conversion pathways, such as glutamate and glucose metabolism, as well as oxidative phosphorylation were among the top upregulated hits. (Fig. 2a). Detailed analysis of major metabolic pathways showed similarities in upregulation of genes that regulate nutrient uptake (glucose transporters, *Sgk1*), glycolysis (*Hk2*, *Aldob*, *Pkm*), gluconeogenesis (*Pck1*, *Fbp1*, *G6pc*), glutamate pathways (*Gpt*, *Got1*, *Glud1*) and fatty acid oxidation (*Ppara*, *Acox1*, *Acox2*, *Pdk4*), in both environmentally (cold exposure) and genetically-induced (*ob/ob*) expanded guts (Fig. 2b). To establish which of these pathways is needed for supporting gut surface expansion and increased caloric uptake, we generated a series of intestine-specific mouse knockouts of rate-limiting enzymes of upregulated pathways, using inducible *villin-CreERT2* line. Target genes were phosphoenolpyruvate kinase (*Pck1*) for gluconeogenesis (Supplementary Fig. 2), hexokinase 2 (*Hk2*) for glycolysis (Supplementary Fig. 3) and glutamate dehydrogenase (*Glud1*) for glutamate oxidation (Supplementary Fig. 4). These specific and complete deletions of the target genes in the intestine neither prevented intestinal adaptations after 30 days of cold exposure, nor altered body weight gain and gut morphology upon HFD feeding (Supplementary Fig. 5).

### Intestinal PPARα is necessary for villi elongation

In search of the mechanisms for the altered intestinal function, we next measured soluble metabolites in jejunum samples of cold-exposed mice. The top enriched metabolic pathways as determined by metabolomics were biosynthesis and metabolism of hydroxyeicosatetraenoic acids (HETE) (Fig. 2c), which are derivatives of arachidonic acid. HETEs activate PPARs, including PPARα^30^. Branched-chain aminoacids and glycolytic and gluconeogenic metabolites were also high after cold exposure. Eicosapentaenoic acid (EPA), another eicosanoid that binds and activates PPARs^31^ was elevated in jejuna of cold-exposed mice (Fig. 2d). The critical regulator of the tricarboxylic acid (TCA) cycle, *Pdk4*, which favours β-oxidation by negatively regulating the enzyme that channels glycolytic carbons into the TCA cycle, was strongly upregulated in cold and *ob/ob* (Fig. 2b). The two main PPAR isoforms expressed in intestines are PPARα and PPARδ, while PPARγ is ten times less abundant (Fig. 2e). Of these, *Ppara* expression is the most abundant in jejunum and the only one that was upregulated by cold exposure and in *ob/ob* mice. Expression of transcriptional regulator *Prdm16* and PPARα co-activator *Rxr* were not changed, while the PPARα target *Nr1h4* (coding for FXR) that regulates fatty acid oxidation was induced in *ob/ob* mice. We used the previously described^32^ *Villin-Cre* x *Ppara lox/lox* mice (*Ppara* I-KO, Supplementary Fig. 6a-f) to study the role of intestinal fatty acid oxidation in gut expansion. We first exposed mice to 6 °C for 30 days to provoke gut expansion (Fig. 2f–l, room temperature controls in Supplementary Fig. 6g-l). The *Ppara* I-KO mice exhibited better oral glucose tolerance, particularly at the early absorption stage (Fig. 2f), which was not the case for intraperitoneal injection (Fig. 2g), and not due to different insulin sensitivity (Fig. 2h). *Ppara* I-KO mice had lower fat mass compared to their controls after 30 days of cold exposure (Fig. 2i), owing to a reduction in both subcutaneous and visceral fat depots. The gut length increased in both *Ppara* I-KO and control mice upon cold exposure compared to room temperature (Fig. 2j, Supplementary Fig. 6j), though I-KO trended toward a smaller perimeter (Fig. 2k). Markedly, *Ppara* I-KO prevented the increase of villi length induced by cold treatment (Fig. 2l, Supplementary Fig. 6l), implying that PPARα is necessary for adaptive elongation of the crypt-villus axis, but dispensable for the longitudinal expansion of the small intestine.

### *Ppara* is the dominant PPAR increased in jejunal epithelial cells upon cold and HFD exposure

PPARα global and liver-specific ablation lead to increased fat accumulation and liver steatosis^26^. *Ppara* expression is induced by a high-fat feeding both transcriptionally and allosterically by fatty acid binding, and PPARα coordinates transition to fasting by activating genes of hepatic fatty acid mobilization, oxidation and ketogenesis^33^. In intestines, PPARα induces FAO as in the liver^34,35^, but its role in relation to global energy homeostasis is less understood. Its expression is highest in the jejunum and decreases progressively along the ileum^36^. Pharmacological activation of PPARα over short-term can inhibit the ingestion of HFD^35,37^, and reduce cholesterol esterification in the intestine^38^. Abrogation of PPARα as the most abundant PPAR isoform (Fig. 2e) does not cause transcriptional compensation of the remaining two isoforms in jejunal tissues (Fig. 2m). As whole intestinal tissue contains many cell types besides epithelial, we characterized spatial distribution of PPARα by sorting intestinal epithelial cells from RT- or cold-exposed *Lgr5-EGFP-IRES-creERT2* mice (Supplementary Fig. 7a-c). *Ppara* and its hallmark target *Pdk4*, but not *Ppard*, are upregulated in response to cold in sorted stem, Paneth, and transient progenitor cells in the crypts, as well as in sorted epithelial cells along the villus (Fig. 2n). Fractionation of intestinal tissue into villus, crypt and muscular-serosal layers confirmed that *Ppara* upregulation is limited to absorptive part of the intestine (Supplementary Fig. 7d). To check if HFD induces *Ppara* expression in the epithelial cells, in parallel to cold exposure, we isolated cells from Lgr5 mice fed HFD on RT, and confirmed predominant upregulation of *Ppara* isoform during HFD in jejunum, particularly in the progenitor cells in the crypts (Fig. 2n, Supplementary Fig. 7d).

### Intestinal *Ppara* KO reduces fat absorption and accumulation during HFD

**Fig. 3:**
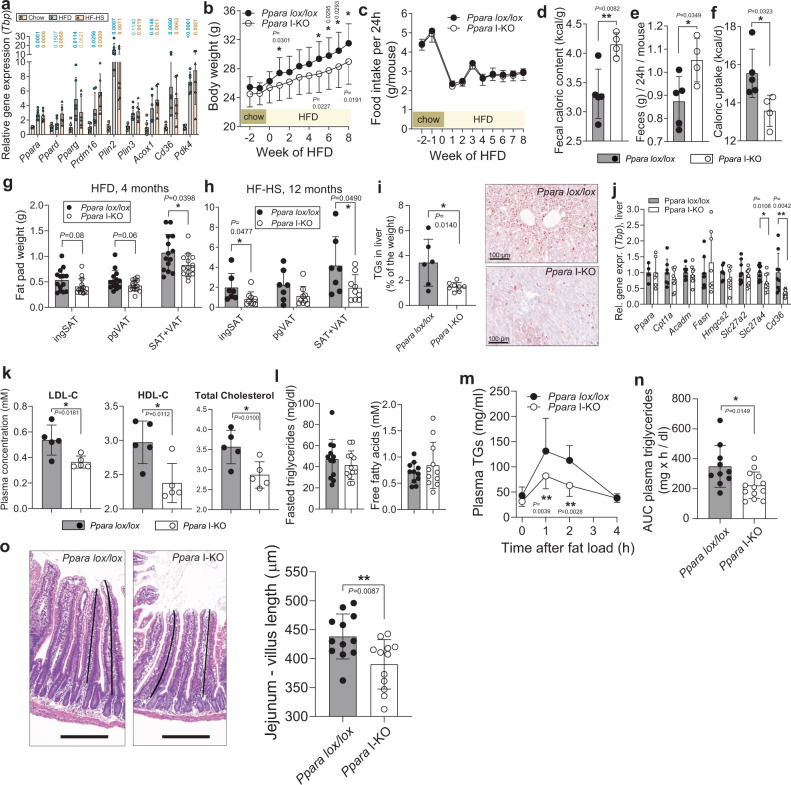
*Ppara* intestinal KO reduces adiposity, caloric uptake and postprandial lipidaemia on HFD. **a** Relative gene expression (qPCR normalized to *Tbp*) in proximal jejuna of WT C57BL6/J mice on ad libitum chow, HFD and HF-HS (*n* = 6 mice per group). **b** Body weight development of *Ppara lox/lox* (*n* = 17), *Ppara* I-KO (*n* = 15) on HFD. **c** Daily food intake per mouse, *n* = 5 cages per group, two mice per cage on HFD. **d**–**f** Caloric density of faecal samples (**d**), daily fecal output (**e**), and total caloric uptake (calories from food − excreted calories) (**f**) in HFD mice, *n* = 5 (lox/lox) or four (I-KO) independent samples, where each sample is a pool of 48 h faeces from two mice in a single cage. **g**–**h** Fat pad weights, after four months of HFD (**g**), *n* = 14 mice per group from two independent experiments, and after 12 months of HF-HS diet (**h**), *n* = 7 (lox/lox) or 9 (I-KO) mice, ingSAT inguinal subcutaneous, pgVAT perigonadal visceral adipose tissue. **i** Triglyceride content in liver, *n* = 6 (*lox/lox*) or eight (I-KO) mice, and representative Oil red O staining of the liver cryosection, scale bar = 100 μm. **j** Relative gene expression (qPCR normalized to *Tbp*) in the liver of *n* = 4–9 (lox/lox) or 6–9 (i-KO) mice pooled from two independent experiments. **k** LDL, HDL and total cholesterol in plasma of fasted mice, measured by Cobas C111 platform, *n* = 5 mice per group. **l** Triglycerides (*n* = 12 mice per group) and free fatty acids (*n* = 11 mice per group) in plasma of fasted mice. **m**, **n** Plasma triglyceride levels after oral administration of 100 μl of olive oil (**m**) and corresponding area under the curve (**n**), *Ppara lox/lox* *n* = 10, I-KO *n* = 13. **o** Representative H&E staining and villi tracing, scale bar = 200 μm, and average jejunum villus length from *n* = 12 mice per group (**o**). **b**–**o** *Ppara lox/lox* and *Ppara* I-KO are male mice on HFD (diet start at age eight weeks), sacrificed at age 25 weeks, if not indicates otherwise. **b**, **c** are representative of three independently repeated experiments, **e**–**o** are pool from two experiments. All data represent mean ± S.D, *P ≤ 0.05, **P < 0.01, ***P < 0.001 of unpaired two-sided *t*-test, confidence level 95%. Source data are provided as a Source Data file.

To investigate the functional role of intestinal PPARα in caloric absorption, we fed C57BL/6 J HFD (60% of calories from fat) or HF-HS diet (60% of calories from fat, 25% from sucrose) for six weeks. Both diets robustly increased expression of *Ppara* in the jejunum, together with its target genes *Acox1* and *Pdk4* (Fig. 3a). *Ppara I*-KO gained less body weight when put on HFD (Fig. 3b), despite the same food intake as controls (Fig. 3c). As energy expenditure did not differ between the controls and *Ppara* I-KO (Supplementary Fig. 8a, b), we measured efficiency of caloric absorption by the intestines using indirect calorimetry of the faecal samples. Caloric density of faeces was higher in *Ppara* I-KO, signifying lower caloric uptake from the ingested food (Fig. 3d–f). After four months of HFD, *Ppara* I-KO mice showed lower total adiposity (Fig. 3g) compared to the control littermates. Similarly, *Ppara* I-KO reduced body weight gain on HF-HS diet, independently of treatment length (Supplementary Fig. 8c). Following 12 months of HF-HS diet, *Ppara* I-KO mice had reduced adiposity (Fig. 3h), and improved oral glucose tolerance (Supplementary Fig. 8d); hence, intestinal PPARα depletion ameliorated the diet-induced obesity independently of the type and length of the high-caloric diet.

As HFD leads to ectopic fat accumulation in the liver, and *Ppara* is most abundantly expressed in the liver, we measured hepatic triglyceride contents. Surprisingly, intestinal *Ppara* KO prevented hepatic steatosis, as triglycerides and neutral lipids were decreased in *Ppara* I-KO mice (Fig. 3i). Liver weight was also reduced (Supplementary Fig. 8f). Therefore, we wondered how the abrogation of PPARα in intestine cause lower fat accumulation in the liver, where PPARα is intact. Quantitative RT-PCR suggested that *Ppara*, and the main genes of hepatic fatty acid oxidation and synthesis were not affected (Fig. 3j). However, the principal regulator of fatty acid transport *Cd36*, and the intracellular long-chain fatty acid transport protein 4 (FATP4, gene *Slc27a4*) were decreased in *Ppara* I-KO livers (Fig. 3j). This hinted to a potential difference in circulating lipids. Indeed, both low- and high- density lipoproteins were reduced in *Ppara* I-KO mice in the fasted state (Fig. 3k), but the levels of fasting plasma triglycerides (TGs) and free fatty acids (FFA) remained comparable (Fig. 3l). To test if the postprandial lipid absorption differed, we orally administered olive oil to fasted mice. Fatty acids are taken up by enterocytes and re-esterified to triglycerides, which are then packaged into chylomicrons. These are then first secreted in lacteal inside the villus, from where they enter the lymphatic system and latter join the systemic blood circulation via the lymphatic thoracic duct. *Ppara* I-KO mice displayed a markedly lower rise in circulating TGs after acute olive oil load (Fig. 3m, n). This difference was not due to altered gut membrane integrity, which remained intact in both *Ppara* I-KO and the controls, as measured by FITC-dextran administration (Supplementary Fig. 8g). TGs from chylomicrons and lipoproteins are hydrolysed by lipoprotein lipase (LPL), whose main sites of expression are endothelial cells in the heart and adipose tissue. Expression of *Lpl* and its co-recruiter *Gpihbp1* were not changed in these organs (Supplementary Fig. 8h, i). These results suggested that attenuated postprandial lipidaemia in *Ppara* I-KO arises from reduced fat absorption in the intestines, rather than increased clearance by the peripheral organs.

### *Ppara* I-KO reduces crypt proliferation

Knock-out of the gut PPARa did not change intestinal length under HFD (Supplementary Fig. 8j). However, consistent with the data from the cold-exposed mice, *Ppara* I-KO villi in jejunum were reduced compared to the control mice (Fig. 3o). These data demonstrate that *Ppara* is necessary but not sufficient for the full growth of a villus in the conditions of increased caloric need (cold) and availability (cold and HFD). We investigated if the effect of PPARα on the villi length may be due to changes in the growth of individual enterocytes, or due to altered cell number along the villi, and found that PPARα deficiency reduced the cell number without affecting their size (Supplementary Fig. 8k, l).

**Fig. 4:**
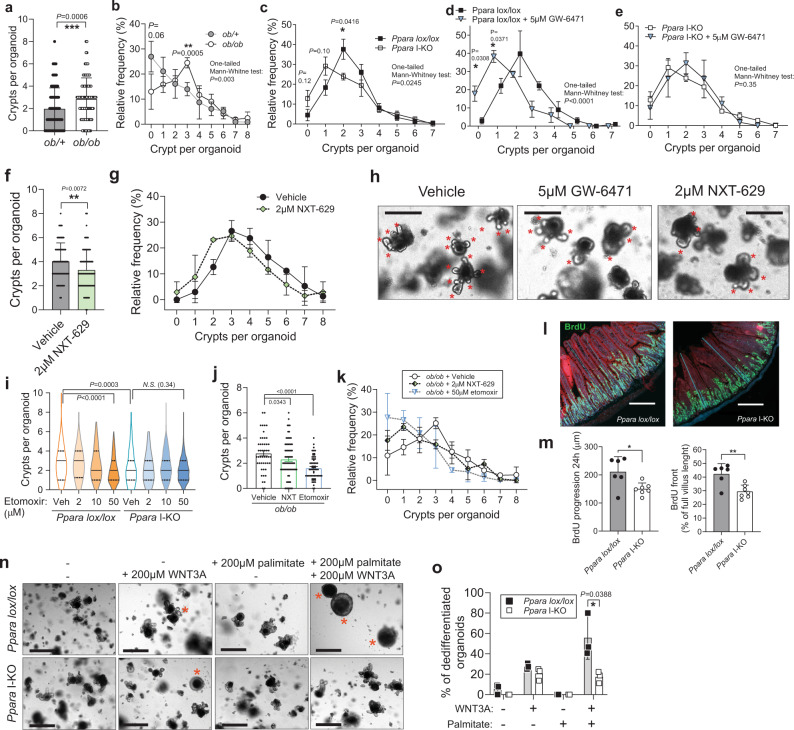
Organoid crypt budding is decreased by PPARα knock-out and inhibition. **a** Number of crypts per organoid in the organoid cultures derived from two *ob/+* and two *ob/ob* mice on chow diet. 120 *ob/+* and 88 *ob/ob* organoids were analysed from 3 wells per group, at day 5, one-tail Mann–Whitney test. **b** Histogram of (**a**) *n* = 3 wells per group. **c** Histogram of organoid distribution according to the number of crypts in the cultures from *Ppara lox/lox* and I-KO mice on chow diet, cultured without inhibitors, pool from two independent experiments and 6 (*lox/lox*) or 5 (I-KO) wells per group, total of 282 (*lox/lox*) or 220 (I-KO) organoids. One-tail Mann–Whitney tests on the histograms refer to the comparison of all organoids between the two groups (as in **a**), and asterisks for difference (two-tailed t-test) between frequencies of distribution on the histograms. **d**, **e** Histogram of organoid distribution by the number of crypts in the cultures from *Ppara lox/lox*, *n* = 3 wells per group (**d**) or *Ppara* I-KO mice (*n* = 5 wells for DMSO, *n* = 3 for GW-6471) (**e**), treated daily with DMSO or 5 μM GW-6471, on day 7. **f** Number of crypts per organoid, on day 9 of daily incubation with 2 μM NXT-629 and its vehicle, from two mice per group, 72 (vehicle) and 69 (NXT-629) organoids analysed, one-tail Mann–Whitney test. **g** Histogram of (**f**). **h** Representative images of organoid cultures from (**d**) and (**f**) on day 9, red asterisks mark crypt outgrowth, scale bar is 200 μm. **i** Violin plot of distribution of organoids per number of crypts in *Ppara lox/lox* and *Ppara I-KO* organoids when incubated with etomoxir, pooled from three wells per group on day 5 in a single experiment, median marked with solid, quartiles with dashed lines. Number of organoid, lox/lox: 251 (vehicle), 84 (2 μM), 169 (10 μM), 162 (50 μM), I-KO: 193 (veh.), 87 (2 μM), 270 (10 μM), 202 (50 μM) j, Number of crypts per organoid from *ob/ob* mice as in (**a**), on day 5 of daily incubation with a vehicle or 2 μM NXT-629, n = 88 (veh.), 127 (NXT), *n* = 201 (etomoxir). **k** Histogram of (**j**). **l**, **m** BrdU staining of jejunum 24 h after injection, with villi counterstained with Evans blue, and thin blue lines indicated measurements of villi and BrdU progression, scale bar is 200μm (**l**), BrdU progression along the villi in μm, and as a percentage of villus length (**m**), *n* = 6 (*lox/lox*), 7 (I-KO) mice per group. **n** Addition of WNT3A to basal ENR medium (EGF, Noggin, R-spondin) induces cystic crypts and organoids (red asterisks), potentiated by addition of palmitate, scale bar is 500μm. **o** Percentage of spherical organoids in the well, *n* = 3 per group, day 7. All data represent mean ± S.D, (except for **I**, as described) *P ≤ 0.05, **P < 0.01, ***P < 0.001 of unpaired two-sided *t*-test, confidence level 95%, except for (**a**, **f**, and **i**), where it stands for one-tail Mann–Whitney non-parametric test. Source data are provided as a Source Data file.

Intestinal surface size depends on stem cell division and subsequent amplification of the progenitors in the intestinal crypts, and the effect of PPARα on the intestinal cell division suggests that PPARα is needed for intestinal crypt proliferation. Therefore, we addressed the role of PPARα in intestinal proliferation using organoid cultures. We isolated crypts from jejuna, and seeded them in matrigel and conditioned medium^19^. To investigate if the amount of budding new crypts from developed organoids is proportional to the in vivo intestinal surface size, we seeded crypts from + */ob* and *ob/ob* mice. Organoids developed from *ob/ob* mice produced more crypts than the controls (Fig. 4a, b). This suggested that crypt-derived organoids in the culture retain proliferative properties of the source intestines. Next, we produced organoids from *Ppara* I-KO and control animals and found reduced budding in the KO organoids (Fig. 4c). We observed similar results by using a selective pharmacological PPARα antagonist GW-6471, where organoids derived from WT mice developed fewer crypts compared to the vehicle-incubated ones (Fig. 4d). To exclude potential PPARα-independent effects of GW-6471, we repeated the experiment in the *Ppara* I-KO mice and found no differences, suggesting that decreased budding is not due to cytotoxicity or non-specific targets (Fig. 4e). Another PPARα antagonist, NXT-629, produced similar inhibition of crypt budding in WT mice (Fig. 4f–h).

To test if the crypt proliferation depends on PPARα-driven fatty acid oxidation, we incubated organoids with etomoxir, an inhibitor of carnitine-palmitoyl transferase 1 (CPT1), necessary for the import of fatty acids into mitochondria for oxidation. We observed a dose-dependent diminishing in the proliferation rates of the crypts isolated from the control mice down to the KO levels. In contrast, we saw no effect on KO organoids (Fig. 4i), suggesting the absence of unspecific off-target effects. PPARα antagonism also reduced crypt budding in conditions of increased *Ppara* expression, both in the cultures from HFD (Supplementary Fig. 9a–d) and from *ob/ob* mice (Fig. 4j, k), whereas the proliferation in *ob/ob* organoids was reduced by etomoxir (Fig. 4j, k). In contrast, PPARα agonism by Wy-16463 increased proliferation on HFD (Supplementary Fig. 9e). Together, these data show that genetic and pharmacologic inhibition of PPARα reduces crypt proliferation capacity and indicate that fatty acid oxidation metabolically supports this process. To test if this is reflected in the proliferation in vivo, we injected mice with 5-bromodeoxyuracil (BrdU). Following 24 h, we detected decreased cell division and turn-over in the *Ppara* I-KOs compared to their control littermates (Fig. 4l, m). The reduced villi, but not small intestinal length in the *Ppara* I-KO mice indicate that PPARα is required for optimal adaptive growth of the crypt-villus axis. We found no difference in the frequency of Paneth, goblet cells or endocrine cells, nor in the most important gut hormones (Supplementary Fig. 10). Transcriptional data from enlarged guts and *Ppara* I-KO show that the markers of intestinal specification cell types and are mostly not affected at the transcriptional level (Supplementary Fig. 11).

A niche for stem cell proliferation is established by WNT proteins, excreted chiefly by Paneth cells, which trigger β-catenin signalling. Overnutrition, but also HFD^15^ and PPARδ^16^ activation increase proliferation in the crypts via Wnt/β-catenin pathway. In our experiments, HFD increased β-catenin levels in whole jejuna, and also in proliferative progenitors (Supplementary Fig. 9f). To examine if PPARα may crosstalk with this pathway, we incubated organoids with exogenous WNT3A. Adding WNT3A favours the formation of round cystic organoids rich in stem cells and progenitors, without differentiation into villus-domain cell types^39^. By adding palmitate to the culture, WNT3A efficiently promoted stemness in the WT littermates, but the stemness was markedly reduced in organoids from the *Ppara* I-KO (Fig. 4n, o). This demonstrates that in the presence of lipids (as during HFD), PPARα is required for fully effective WNT/β-catenin signalling, and stem cell division in the crypt leading to full villus growth.

### PPARα antagonism reduces absorption in human intestinal biopsies independently of fatty acid oxidation

**Fig. 5:**
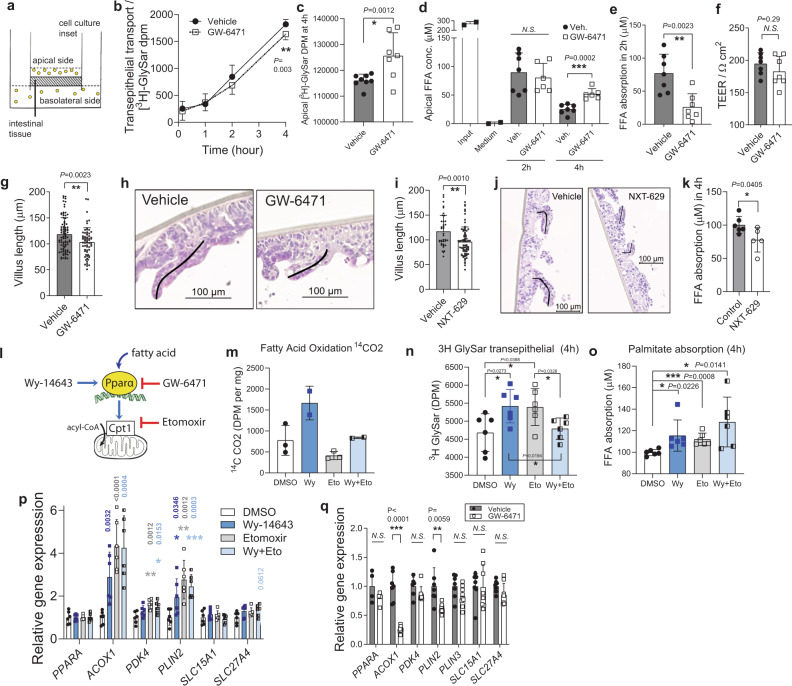
PPARα antagonism reduces fatty acid uptake and villi length in human intestinal biopsies. **a** Graphical representation of the human epithelial biopsy culture. **b**, **c** Basolateral (**b**) and apical radioactivity (**c**) of dipeptide [^3^H]GlySar, *n* = 7 per group of epithelial biopsy insets cultured seven days with PPARα antagonist 5 μM GW-6471 or DMSO. **d**, **e** Apical concentration of free fatty acids (**d**) and calculated palmitate absorption between 2 and 4 h, *n* = 7 (vehicle), 6 (GW-6471) biopsy insets (**e**). **f** Trans-epithelial electrical resistance at the end of the experiment, *n* = 7 (vehicle), six (GW-6471) biopsy insets. **g**, **h** Full villi lengths *n* = 88 (vehicle), 63 (GW-6471), pooled from *n* = 5 (vehicle), four (GW-6471) biopsy insets after nine days of treatment (**g**) and representative H&E staining with villi tracing (**h**). **i**, **j** Full villi lengths after nine days of incubation with PPARα antagonist 5 μM NXT-629 (*n* = 70 villi), or vehicle (*n* = 38 villi), pooled from four cell culture insets per group (**i**) and representative H&E staining of villi (**j**). **k** Palmitate absorption in 4 h, from *n* = 6 (vehicle) and four (NXT-629) insets per group, calculated from apical FFA concentration measurements. **l** Graphical representation of the mechanism of pharmacological PPARα agonism (Wy-14643) and antagonism (GW-6471), and of the fatty acid oxidation inhibitor etomoxir. **m** Fatty acid oxidation in human epithelial samples measured by scintillation counting of [^14^C]CO_2_ that is released by in vitro β-oxidation of [^14^C]-palmitate, *n* = 2 (DMSO, etomoxir), three (Wy-14643, Wy-14643+etomoxir). **n**–**p** Basolateral radioactivity of dipeptide [^3^H]GlySar after 4 h of transport (**n**), palmitate absorption in 4 h, calculated from apical FFA measurements (**o**) and relative qPCR gene expression in epithelial biopsies, normalized to *Tbp* (**p**) after 10-day inhibition with DMSO, 4 μM Wy-14643, 50 μM etomoxir, and Wy-14643 + etomoxir, *n* = 6 biopsy insets per group. **q** Relative qPCR gene expression after GW-6471 treatment, *n* = 7 (vehicle), eight (GW-6471) insets per group. **b**–**h** and **q** are pools from two independent experiments. All data represent mean ± S.D, *P ≤ 0.05, **P < 0.01, ***P < 0.001 of *t*-test confidence level 95%. Source data are provided as a Source Data file.

To establish the relevance of PPARα-mediated villus growth in humans, and elucidate its role in promoting nutrient absorption, we used commercial human intestinal epithelia biopsies mounted to cell culture insets (MatTek EpiIntestinal 3D tissue model, Fig. 5a)^40^. These tissues obtained from healthy donors maintain crypt-villus polarity native structure and physiology up to several weeks. We applied small volumes of tissue medium with vehicle or PPARα antagonist GW-6471 to the apical side of the epithelium, thus mimicking the interface of the native brush border and the lumen. A five-day incubation with GW-6471 moderately reduced the uptake of non-metabolizable dipeptide glycyl-sarcosine (GlySar), as measured by its concentration in the basolateral medium (Fig. 5b) and its decrease in the apical medium (Fig. 5c). The reduction (~50%) of palmitate uptake was even stronger (Fig. 5d, e). These effects were not due to any alteration in permeability of the intestinal barrier in the human biopsies, the integrity of which remained intact (Fig. 5f). Remarkably, in samples incubated only 8-days with GW-6471 the villi shortened (Fig. 5g, h), in agreement with the decreased organoid proliferation upon treatment with the PPARα antagonist, and with the in vivo data using the *Ppara* I-KO mice. Similar reduction in fatty acid uptake and villi length was obtained with the alternative PPARα antagonist NXT-629 (Fig. 5i-k), indicating that the effects of PPARα antagonism are independent of the choice of the drug.

We next investigated how PPARα regulates fatty acid absorption and villus growth. PPARα regulates fatty acid β-oxidation (FAO), as well as aspects of lipid transport and droplet formation^33,41^. To determine if intestinal FAO is necessary for lipid absorption, we treated human epithelial cultures with etomoxir (Fig. 5l), which inhibited FAO (Fig. 5m). In turn, PPARα activator Wy-14643 increased FAO, which was prevented by co-incubation with etomoxir (Fig. 5m). The activator increased both dipeptide and palmitate uptake (Fig. 5n, o). Paradoxically, etomoxir mimicked the effect of the activator. Co-incubation of Wy-14643 with etomoxir not only failed in preventing the increase of the acute palmitate uptake, but further slightly raised it, which was not the case for the dipeptide uptake (Fig. 5n, o). This suggests that the decrease of lipid uptake observed in *Ppara* I-KO and after PPARα antagonism may not be due to decreased FAO (Supplementary Fig. 9g–i, Supplementary Table 3, Fig. 5m), but through some other PPARα-mediated process.

### PPARα coordinates lipid absorption in the gut by regulating lipid droplet expansion

Enterocytes take up dietary fat in form of free fatty acids and monoacylglycerols, which are formed from triglycerides^42^. CD36 is a key intestinal fatty acid transporter, while SLC27A4 is the main fatty acid-binding protein that channels the fatty acids through enterocytes and activates them for future metabolism^43^. In enterocytes, free fatty acids and monoacylglycerols are re-esterified into triglycerides and exported to the blood in chylomicrons, while a fraction remains in enterocytes as cytosolic LDs (especially after a fat-rich meal and in diet-induced obesity). Formation of cytosolic LDs depends on a number of cytosolic LD-associated proteins, including perilipin-2 and 3^44^. In addition to increasing the expression of *Acox1* and *Pdk4*, PPARα activation in tissue cultures also enhanced *Plin2* (Fig. 5p), a lipid droplet surface protein in the intestines. Etomoxir also increased *Plin2*, as noticed earlier^45^. On the other hand, peptide and fatty acid transporters *Slc15a1* and *Slc27a4* showed no (or only marginal) increase in expression. Nonetheless, inhibition of PPARα reversed these effects (Fig. 5q).

**Fig. 6:**
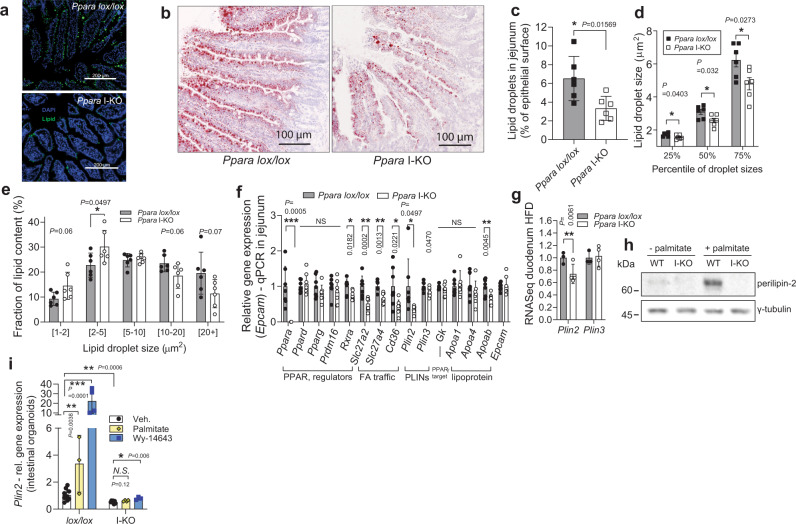
PPARα ablation reduces LD size and amount, and suppresses PLIN2 in small intestine. **a** Neutral lipids in cytosolic LDs in proximal jejunum stained green by LipidTOX, one hour after 100 μl olive oil gavage. **b** Representative Oil red O staining of jejunum cryosection 1 h after olive oil gavage. **c**–**e** Cytosolic LD area as a percentage of total epithelial area (**c**), lipid droplet size distribution (**d**) and contribution of different cytosolic LD sizes toward total lipid area (**e**), quantified from Oil red O stainings of proximal jejuna, 1 h after olive oil load, *n* = 6 female mice per group (each sample is the average of three sections. **f** Relative qPCR gene expression, normalized to *Tbp*, in jejuna of fasted mice on HFD, *n* = 7 per group. **g** Relative RNA sequencing gene expression of *Plin2* and three in duodenum of Ppara mice on HFD, *n* = 3 (*lox/lox*), four (I-KO mice). **h** Western blot against perilipin-2 in the organoids from *Ppara lox/lox* or I-KO, treated with 0.4 mM palmitate: BSA, or BSA. **i** Expression of *Ppara* target genes in the crypts as in (**h**), pool of two experiments, relative to *lox/lox* vehicle group, *n* = 7 for vehicle groups, 3–4 for treatments. All data represent mean ± S.D, *P ≤ 0.05, **P < 0.01, ***P < 0.001 of unpaired two-sided *t*-test confidence level 95%. Source data are provided as a Source Data file.

To investigate if there is a change in lipid uptake and droplet formation in the intestines, we orally administered 100 μl of olive oil to mice that were fasted overnight. After one hour, mice were sacrificed and the sections of jejuna were treated with neutral lipid stains LipidTOX or Oil Red O (Fig. 6a, b). Quantification of lipid droplets in the intestinal epithelium revealed that the enterocytes of *Ppara* I-KO mice had diminished cytoplasmic lipid content, compared to epithelium of the control littermates (Fig. 6c). The lipid droplets of the *Ppara* I-KO mice were smaller than those of the controls (Fig. 6d), and these smaller droplets contributed more to total lipid droplet area than large droplets; in contrast to the controls (Fig. 6e). These observations are consistent with the attenuated postprandial lipidaemia in *Ppara* I-KO mice at 1 to 2 hours after acute fat load (as in Fig. 3m). In *Ppara* I-KO jejuna, *Cd36*, *Slc27a2* and *Slc27a4* expression was decreased, whereas genes critical for glucose and peptide absorption, the re-esterification of absorbed fatty acids, packaging in chylomicrons, and mitochondrial content were not affected (Fig. 6f, Supplementary Fig. 9k, Supplementary Fig. 10m). In contrast, *Plin2* expression was strongly suppressed in *Ppara* I-KO, both at mRNA and protein level (Fig. 6f-h). PLIN2 (also known as adipophilin, ADRP) is critical for LD formation and stability, and its absence hampers the ability to form lipid droplets in many tissues^46–48^. Therefore, we further investigated the PPARα-dependant response of PLIN2 to palmitate load in intestinal organoids from *Ppara* I-KO and control mice. Palmitate increased PLIN2 protein levels over 8-fold in WT organoids. This increase was abolished in *Ppara* I-KO derived organoids (with less than 1% remaining, Fig. 6h), demonstrating a near-complete depletion of PLIN2 upon PPARα deletion. Conversely, palmitate enhanced the *Plin2* transcript for over 4-fold in WT organoids, while PPARα agonism led to over 20-fold increase of the *Plin2* levels. Both palmitate and PPARα agonism had no effect on *Plin2* expression in *Ppara* I-KO organoids (Fig. 6i), showing that the increase of PLIN2 after pharmacological activation of PPARα, and importantly upon palmitate stimulation, is PPARα-dependant. Collectively, these findings suggest that lack of PPARα delays fat absorption through the intestine and that, at least partially, this is mediated by downregulation of perilipin 2, necessary for enterocyte lipid droplet formation.

## Discussion

Our study addresses a fundamental question on the metabolic triggers and mechanisms regulating the plasticity and function of the intestinal lining. The work shows that the intestinal functional and morphological alterations depend on the food amount. Using multiple genetic models, and systematic in vivo approaches, we demonstrate that while several energetic pathways are dispensable, the intestinal PPARα-mediated lipid metabolism is of key importance for the adaptive villi lengthening and increased absorptive function in mice and human intestinal biopsies. This work proposes a concept in which the intestinal plasticity allows optimisation of caloric uptake from the consumed food, by coupling energetically costly intestinal epithelial maintenance to food availability.

Gut reshaping involves elongation, villus growth, microvilli extension, and upregulation of uptake transporters, though not all of them need to be employed equally and at the same time. These processes require energy resources. While our data show that gut expansion mobilizes glycolysis, gluconeogenesis and glutamate utilization pathways; there is a robust capacity for compensation in case one of them is affected. However, we found that PPARα is indispensable for adaptive villi growth and increased lipid transport. While fatty acid oxidation that is orchestrated by PPARα was needed for crypt-villus growth, its inhibition was insufficient to prevent intestinal lipid transport. Instead, our data suggest an alternative pathway, where the intestinal lipid droplet formation is reduced following PPARα depletion, coupled to a marked reduction of PLIN2, a key mediator of lipid droplet formation and growth^48^. In DIO, PLIN2 is upregulated, forming ring-like structures around cytosolic lipid droplets as they grow^44,49^, and *Plin2* deletion affects lipid absorption in intestines and ameliorates DIO^49^. In our experiments, PPARα deletion induced complete blunting of the *Plin2* expression, and fully prevented its elevation driven by an increase in the fatty acid palmitate, which seemed sufficient to suppress the total TG absorption in line with data from the global *Plin2*-KO mice^49^. This notion can be further supported by the current understanding of cytosolic lipid droplets representing a transitional pool of lipids that optimizes absorption during food consumption^50^. Therefore, the reduced lipid uptake following PPARα inhibition may be a result of three complementary effects: decreased intestinal surface due to shorter villi, downregulation of fatty acid transporters on the apical surface of brush border, and reduced expression of *Plin2* in the enterocytes. All three layers of regulation were considerably lower with inhibition or knock-out of *Ppara*, explaining the potent reduction of lipid uptake. These mechanisms also affect sugar and peptide transport, though ex vivo uptake assays and the respective transporter expression data suggest that their uptake is attenuated to a lesser extent than fatty acids.

We found no effect of PPARα deletion on the intestinal lipoprotein assembly machinery, but rather a decrease of fatty acid uptake, packaging and export, independent of fatty acid oxidation status. Earlier reports suggested that docosahexaenoic acid^51^ and benzafibrate^52,53^, activators of PPARα and fatty acid oxidation, attenuate postprandial hyperlipidaemia in vivo and lower basal TG secretion in Caco-2 enterocyte cultures. It is important to note that these studies used exclusively pharmacological approaches, did not measure TG build-up and export in enterocytes after fat load, nor investigated for morphological changes on enterocytes, or contributions from other organs where the pharmacological PPARα activation would play a role. In line with our findings, it is known that ISCs are sensitive to dietary clues, and fatty acids provided by HFD increase ISC proliferation. This was recently attributed to activation of PPARδ^16^, an abundant PPAR isoform in the crypt region, and to PRDM16, a transcriptional regulator of FAO^54^. However, deletion of PPARδ did not alter villi or gut length^16^. Moreover, we found no or very small increase in its expression in any of the enlarged intestines, compared to the PPARα. As PPARδ is more dominant isoform in duodenum, and PPARα in jejunum (Supplementary Fig. 6e, g), it is possible that the two isoforms regulate self-renewal of different parts of intestine, and both PPARα and PPARδ deficiencies attenuate response to WNT3/β-catenin signalling, which reduces stem cell division and cell cycling. However, in contrast to *Ppara*, the *Ppard* I-KO triggers increased fat gain during HFD^55^, indicating that the mechanism of fat handling is distinct in the two knockouts. The contribution of PPARα in the intestinal response to HFD was not addressed in these studies, but Hmgcs2 is a well-known target of PPARα^33,41^, while PRDM16 is the upstream regulator of PPARα and other genes of FAO, especially the upper intestine. Fat-rich ketogenic diet activates HMGCS2 (the rate-limiting enzyme of ketogenesis) in ISC, which stimulates Notch signalling to promote ISC self-renewal^56^. In line with this, our data suggest PPARα as an indispensable mediator of intestinal homeostasis and cell division in jejunum, likely downstream of PRDM16.

It is important to note that various genetic and dietary models can have pleiotropic effects, which may contribute to the correlations observed between food intake and gut surface area. While our study points to the critical contribution of the food amount, it does not exclude that certain food components^29^ can affect the intestinal surface and function independently, or in concert with the food amount. Moreover, the transcription programs orchestrated by PPARα are indispensable for the villi growth in presence of dietary lipids, however, the direct link between the lipid metabolism and the food amount pends further investigations. As recently reported, PPAR and RXR signalling is involved in the complex transitions between early regeneration and latter differentiation in intestinal organoids^57^, and knock-down of RXR, a PPAR co-factor, reduced enterocyte generation, but increased the regenerative capacity. Intriguingly, while PPARα coordinates lipid metabolism and is upregulated by lipid-rich diets, there is no direct correlation between amount of ingested lipid and intestinal surface. Indeed, palmitate and HFD increase crypt formation and stem cell division without resulting in expansion of the absorptive surface (our data and^16^). All this shows that PPARα is essential, but not sufficient for gut surface increase, and that additional mechanisms are necessary to release the break on the intestinal surface expansion. In the case of overeating, this might involve activation of other energy pathways in concert with PPARα, and sensing of mechanical pressure in the intestines.

Mucosa thickening is a hallmark of intestinal adaptations that follow intestinal resection. It is, at the molecular level, a poorly understood process that includes bowel lengthening, growth of villi, and induction of transporter expression^58^. The PPARα-driven mechanism of villus growth could explain why fatty acids such as palmitate or linoleate are the most effective dietary therapy that promotes mucosa re-growth after bowel resection^59,60^, a process that can be further potentiated by feeding prostaglandins^61^. Fatty acids and eicosanoids are strong inducers of PPARα. Conversely, disruption of PPARα and fatty acid oxidation is found in celiac disease, a condition characterized by shortened villi and malabsorption^62^.

Hepatic steatosis and increased adiposity are major risk factors for the development of metabolic syndrome. PPARα ablation in the liver and adipose tissue facilitates fat accumulation in these tissues by reducing levels of fatty acid oxidation^26^. Unexpectedly, *Ppara* deletion in the intestine lowers fat uptake in the liver and fat. Intestinal lipid droplet formation and trafficking regulated by PPARα, therefore, represent important rate-limiting steps in systemic lipid metabolism. Inhibition of this process moderates the excursions of plasma triglycerides after a lipid-rich meal. If pharmacological inhibition of PPARα or lipid droplet formation could be limited to intestines, this would serve as an option for reducing fat absorption and hyperlipidaemia, but also total caloric uptake. As a whole, the remarkable gut plasticity that permits gut and villi to be shrunk or enlarged is, therefore, an asset as a reversible alternative to gastric bypass surgery, or other interventions that aim at reducing body weight gain and obesity-related complications.

## Methods

### Animals

C57BL/6 J, *ob/+* and *ob/ob* animals were purchased from Charles River France. *Hk2 lox/lox* mice (B6.129P2(Cg)-Hk2 < tm1.1Uku) were purchased from EMMA; *Pck1 lox/lox* mice (Pck1-tm1) from MMRRC; while *Glud1 lox/lox* were generated and described before^63,64^ (Glud1tm1.1Pma, MGI:3835667). Genotyping protocols and primers for Hk2, Pck1 and Glud1 mice were taken from respective suppliers. They were all crossed with *villin-CreERT2* mice (B6.Cg-Tg(Vil1-cre/ERT2)23Syr/J), which were gift from Eduard Battle (IRB Barcelona) with permission from Sylvie Robine (Institute Curie). Mice were kept lox/lox homozygous and Cre transgene heterogeneous. Mice crossed with *villin-CreERT2* line were administered in total 3 intraperitoneal (i.p.) tamoxifen injections (10 mg per ml of corn oil, 2 mg per 25 g of body weight), one every other day, starting at of 7 weeks, one week before the start of the experimental treatments. Efficiency of knock-out was confirmed by PCR in whole intestinal tissues and in the sorted intestinal crypt cells. *Villin-Cre* mice (B6·Cg-Tg(Vil1-cre)1000Gum/J) were purchased from Jackson Laboratory, crossed with *Ppara lox/lox*, and maintained by *Ppara loxP/loxP x Ppara lox/lox; Cre* breeding. Deletion and tissue specificity were checked as previously characterized^32^. Lgr5-EGFP-IRES-creERT2 mice were purchased from Jackson Laboratory. All mice were on predominantly C57BL6/J background. All mice were bred, housed and experimented with in SPF facilities, except Ppara mice, which were bred and experimented with in the conventional zone. Control mice for all intestinal KO mice were always their lox/lox littermates, also injected with tamoxifen (where applicable). All mice were kept on 12-h light (7am–7pm)/dark cycle on 23 °C, while cold exposure was on 6 °C, in the light and humidity controlled (40%) chambers. Cold-exposed mice were always kept two per cage, without cotton pads and houses, separated by the genotype, while HFD and HF-HS mice were kept 2–4 mice per cage, with the enrichment, separated by the genotype (the cohorts for the food intake and faeces measurements), or mixed (in the repeated experiments). All mice were males, unless specified otherwise in a Figure legend, and in vivo measurements were not blinded. All procedures were approved by the Geneva cantonal and the Swiss federal authorities for animal care and experimentation under GE/84/19 and GE/146/19. Animal research was conducted according to the Swiss regulations (Federal Animal Welfare Act) and guidelines (RESAL - Lemanic Animal Facility Network).

### Diets

The different foods are summarised in Supplementary Table 2. *“Standard chow”* was, if not indicated otherwise, RM3-E-SQC from Special Diets Service (SDS, product code 811181, 4.2% weight of crude fibre, 11.4 MJ/kg of ME). *High-fat (HFD) diet* (60% kJ from fat, 6.0% of weight crude fibre, 21.4 MJ/kg of metabolizable energy [ME]) was from ssniff (Germany), E15742-34 (“blue pellets”). *High-fat high-sucrose (HF-HS) diet*, also known as Surwit diet (59% kJ from fats, 26% kJ from carbohydrates, 0.1% of weight crude fibre, 22.8 MJ/kg of ME), was from ssniff, EF D12331 mod. (“orange pellets”). *Energy-reduced food* was from ssniff (SM – Energy reduced, V9631-S710), and had energy density reduced by 40% compared to chow, mainly by replacement of starch with fibre (38% kJ from carbohydrates, 51% kJ from protein, 11% kJ from fat, 17.3% of weight crude fibre, 7.7 MJ/kg of ME). *HFD no fiber diet* in Supplementary Figure 1d, h, i, and Fig. 2n is from SAFE diets (France), 260 HF, product code U8978 Version 19, (60% kJ from fat, 0% fibre, 23.1 MJ/kg of ME) *HFD added fibre* (code HF150, SAFE) is nearly identical diet, with added 1.4% cellulose (22.2 MJ/kg of ME). Additionally, chow diets from SAFE diets (SAFE-150) and sniff (V1554-703) were used in Supplementary Fig. 1e–i, and chow SAFE-150 in Figs. 2n and 4. All foods were γ-irradiated.

### Animal procedures and measurements

Male 14 to 18-week-old Ppara mice were placed in the calorimetric cages (Labmaster, TSE, BadHomburg, Germany) to measure food intake, locomotor activity, oxygen consumption and CO_2_ production during seven days; energy expenditure was normalized to lean mass. For acute fat load test, mice were fasted for 3 h and gavaged with 100 μl of olive oil (Sigma-Aldrich). Blood samples during oral and intraperitoneal glucose tolerance tests (OGTT, IPGTT) and acute fat load test were collected from the tail vein in EDTA coated tubes (Sarstedt, CB 300 K2E). For OGTT or IPGTT, mice were fasted 12 h overnight and gavaged or injected, respectively, with 2 mg of glucose per g of body weight. For bomb calorimetry of the faeces, 48-hour faeces were collected from the cage bedding (2 mice per cage), vacuum dried, pulverized to the fine powder and analysed in the calorimeter (Parr, 6100, USA). Consumed energy was calculated by formula: digestible energy (kcal) = food intake (g) x food caloric density (kcal/g) − faeces weight (g) x faeces caloric density (kcal/g).

### Tissue and blood processing and analysis

Before sacrifice, mice were fasted 5 hours (unless specified otherwise). Blood collection. 500 μl of blood was taken from terminally anesthetized mice in tubes with 11 μl of 0.5 mM EDTA, 4 μl of aprotinin (1.3%) and 4 μl of DPP-IV (10 mM) and plasma stored at −80 °C. GLP-1, GIP, glucagon and ghrelin were measured by Bio-Plex Pro Mouse Diabetes panel (#17003661), from plasma diluted 1:4 according to instructions, on Bio-Plex 200 Luminex platform (Biorad). Triglycerides were measured by Trig/GB kit (Roche), free fatty acids by NEFA-HR kit (Wako). Intestines were carefully unfolded and samples taken ensuring that they are from the same sites between different mice. *RNA isolation and qPCR*. RNA from tissues and cell cultures was isolated with TRIzol (Life Technologies), cDNA synthetized by High-Capacity cDNA Reverse Transcription Kit (Applied Biosciences). Transcript levels were measured by quantitative RT-PCR using SYBR Green, on the instrument LightCycler 480 (Roche), and the levels were normalized to *Tbp or Rplp0*, as indicated in the legend, or to epithelial marker *Epcam* (for the organoid cultures). The primers used for the qPCR are provided in the Supplementary Table 1. *Metabolomics* analysis was done as described earlier^3,65^. Complete metabolomics measurements and statistical methods are provided in the Supplementary Data 1 of this paper. *RNA Sequencing*. RNA was isolated by TRIzol regent. RNA sequencing for RT and cold groups (sequencing 1), and *ob/ob* and C57BL6/J control (sequencing 2) were done by iGE3 Genomics Centre of University of Geneva, with methodology detailed earlier^3^.

Three samples were submitted per group; each being a pool of two equal amounts of RNA isolated from proximal jejunum. The reads were mapped with the TopHat v.2 software to the UCSC mm10 reference; on new junctions and known junctions annotations. Biological quality control and summarization were done with RSeQC-2.3.3 and PicardTools1.92. The differential expression analysis was performed with the statistical analysis R/Bioconductor package EdgeR v. 3.4.2, for the genes annotated in mm10. Briefly, the counts were normalized according to the library size and filtered. The genes having a count above 1 count per million reads (cpm) in at least 3 samples were kept for the analysis. The differentially expressed genes tests were done with a GLM (general linear model) with a negative binomial distribution. Normalized counts are shown. For comparison on the graphs in Fig. 2, RT and *ob* controls were merged together in a single WT group. For *Ppara* I-KO (*n* = 4) and *Ppara lox/lox* (*n* = 3), duodena from male mice on 8-week HFD were used. Western blot. Tissue and crypt cultures were homogenized with the tissue lyser in the RIPA buffer with protease and phosphatase inhibitors, and cleared by centrifugation, according to standard techniques, and separated by SDS electrophoresis on 10% polyacrylamide gel. Western blots were probed with antibodies against: PLIN2 (guinea pig, 1:1000, MyBioSource), γ-tubulin (1:5000, Sigma Aldrich #T6557), GAPDH (1:1000, abcam), PCNA (1:500, origene, TA309795), PEPCK (1:1000, sc-74825, Santa Cruz), GDH (1:1000, Cell Signalling).

### Microscopy

Tissues for haematoxylin and eosin staining (H&E) and immunohistochemistry were saved in 4% paraformaldehyde (PFA), and for electron microscopy in 2.5% glutaraldehyde in cacodylate buffer. For lipid droplet visualization and quantification, the liver and proximal to middle jejunum samples were frozen on dry ice, cryosectioned, fixed briefly in 4% PFA and treated with Oil Red O with haematoxylin counterstain, or with LipdTOX HCS LipidTOX™ Green Neutral Lipid Stain (Invitrogen) and DAPI (Vectrashield). Quantification was done from Oil Red O staining, in FIJI ImageJ software 1.51 by thresholding stained scans in red channel, then automatic counting of lipid droplets and measuring their area, then normalizing to total epithelial tissue area (measured by thresholding it for entire tissue). Liver triglycerides were isolated by Folch method and resuspended lipids quantified by Trig/GB kit (Roche). *Villi analysis*. H&E sections of proximal jejunum were scanned by Axioscan.Z1 platform (Zeiss) with 20x objective. Perimeters were traced around complete sections, following outer edge of submucosa. Villi lengths were measured in ZEN 2 software by tracing intact, full-length villi from the beginning of the villus domain until the top. Broken, cross-cut, or degraded villi were not measured. In total, 5–10 villi per section from 2–3 sections per jejunum were measured, and the average length reported. Microvilli were imaged in the samples from the proximal jejuna by the transmission electron microscope Morgagni (FEI Company, Eindhoven, Netherlands) at EM Core Facility of University of Geneva. 40-80 electron micrographs per section, taken from the middle part of multiple villi, and 6 representative microvilli along the brush border of the micrograph were measured in iTEM software (ResAlta). *Immunofluorescence*. Antigen retrieval was in 10 mM citrate pH 7.0 buffer, 15 min in microwave, permeabilization in Triton X-100 0.1%, primary and secondary antibodies were diluted in PBS, 1% BSA, 0.1% Tween-20, and washed with 3x with PBS. Antibodies (1:200): LysC (sc-27958, Santa Cruz), anti-BrdU (ab6326, Abcam), chromogranin A (sc-1488, Santa Cruz), Alcian blue was used as previously described^3^. For histological experiments, researchers were blinded to group allocation during processing and quantification of the sample through labelling of samples by the numeric codes.

### Crypt isolation and organoid culture

Intestinal crypts were isolated and the cultured as described before^19^. In brief, 4–5 cm segments of proximal jejuna were gently stripped from villi, washed with PBS, then incubated 30 min, at 4 °C in PBS with 2 mM EDTA. Crypts were released by pipetting up and down, counted and seeded 75 crypts in 20 μl of Matrigel (Corning 356231 growth factor reduced) per well of flat-bottom 48-well plate. After solidification, 300 μl of ENR (EGF, noggin, r-spondin) medium was added in which the organoids were cultured: Advanced DMEM/F12 medium, supplemented with EGF 40 ng/ml (Peprotech), recombinant murine Noggin 200 ng/ml (Peprotech), R-spondin (a gift from Gerald Schwank, University of Zurich; 5 ml of R-spondin conditioned medium from 293T-HA-RspoI-Fc cell line per 50 ml of the culture medium), 1 μM N-acetyl-L-cysteine (Life technologies), 1X N2 (Life Technologies), 1X B27 supplement (Life Technologies). For WENR medium, murine WNT3A (Peprotech) was added to ENR in the concentration indicated. Treatment with drugs started on second day after seeding with 2 μM GW-6471 (Sigma-Aldrich), 2 μM NXT-629 (MedChemTronica), 4 μM Wy-14643 (Sigma-Aldrich), 50 μM etomoxir (Lucerna), 200 mM palmitate:BSA complex, or appropriate vehicle controls (DMSO, fatty acid-free BSA) in the conditioned medium, and media were changed every two days. The plates were maintained at 37 °C, 5% CO_2_, in humidified incubator. Plates were scanned in z-stacks on days 5, 7 and/or 9 on ImageXpress platform, and crypts were counted blindly in ImageJ. 40–60 organoids were counted per well, and 2–3 wells condition. Crypts were harvested for RNA or protein isolation on days 7–10 by dissolving matrigel in ice-cold PBS.

### Flow cytometry (FACS)

Crypts from Lgr5-GFP mice were isolated as above, and suspension of villi in PBS was obtained by gently scraping longitudinally opened section of proximal-to-mid jejunum by microscope cover glass. Cell suspension was spun down, resuspended in 1 ml of undiluted TrypLE Express (Invitrogen) + 100 µl of DNase I (10 U/µl, Roche), then incubated in a 32 °C thermobloc for 2 min, without shaking, and placed on ice. Pelleted cells were washed once in Advanced DMEM/F12, and incubated 30 min on ice in antibody mix of 1 μl of each CD45-PE (eBioscience, 30-F11), CD31-PE (Biolegend, Mec13.3), Ter119-PE (Biolegend, Ter119), CD24-Pacific Blue (Biolegend, M1/69), and EPCAM-APC (eBioscience, G8.8) in 1 ml of Advanced DMEM/F12. Cells were washed twice in PBS and filtered through 70 μm mesh. 7-aminoactinomycin D (7-AAD) was added to exclude dead cells. The cells were sorted on BD FACS ARIA Fusion, with software BD FACS Diva Software version 8.0.2. CD31^−^ CD45^−^ Ter119- 7-AAD + were excluded, others isolated as follows: *intestinal stem cells*: Lgr5-EGFP^high^ EpCAM^+^ CD24^low/−^; *transiently amplyfing progenitors***:** EGFP^low^ EpCAM^+^ CD24^low/−^; *Paneth cells:* CD24^high^/Sidescatter^high^ EGFP^−^ EpCAM^+^; *epithelial cells from villus*: EpCAM^+^. FACS plots have been produced by FlowJoTM version 10.7. Cells were sorted in RTL solution (Qiagen). RNA was isolated from the pellets with RNeasy® Micro Kit (Qiagen) according to the manufacturer’s instruction for isolation from a cell suspension, and cDNA synthesized by High-Capacity cDNA Reverse Transcription Kit (Applied Biosciences).

### Human epiIntestinal cultures

Human intestinal 3D reconstituted epithelial biopsies were obtained from a commercial supplier MatTek Life Sciences Europe (Bratislava, Slovakia) (MatTek EpiIntestinal™). MatTek obtained tissue samples from accredited institutions after informed consent of the donor or next of kin for use of cells or tissues for research purposes. Samples were collected and prepared in accordance with applicable regulations and guidelines including General Data Protection Regulation (Regulation (EU)2016/679), are fully anonymized, and the study was conducted according to the Declaration of Helsinki on biomedical research. Biopsy cultures were maintained in proprietary medium supplanted on day 2 on apical side with the inhibitors in the concentrations described in the previous section, and renewed every day. For glucose uptake assay: 30 mM D-glucose spiked with 0.1 μCi/ml of D[14 C]-glucose (specific activity 55 mCi/mmol, Anawa, Switzerland) was applied to apical side of a tissue inset. For dipeptide uptake assay: 20 μM of glycyl-sarcosine (Sigma-Aldrich), spiked with glycyl-[2-^3^H]-sarcosine, and apical and basolateral medium sampled at 0, 1, 2 and 4 hours. For palmitate absorption, 400 μM of palmitate: BSA complex was applied (together with the treatment antagonist or agonist) to apical side (150 μl total volume), and 10 μl of apical compartment was sampled at time 0, 2 and 4 h. Free fatty acids in sampled aliquots were measured by NEFA-HR kit (Wako). For fatty acid oxidation test, the tissue membrane was incubated 2 h with 200 μM of L-carnitine hydrochloride, 200 μM of palmitic acid, spiked with 0.5 μCi/ml of palmitic acid (Anawa, [1-^14^C] / s.A. 50-60 mCi/mmol, Conc. 0.5 mCi/ml), at 37 °C. CO_2_ released was captured on the filter paper soaked in 1 M NaOH and placed in the cap tube. Filter paper was put in 3 ml of the scintillation liquid (PerkinElmer Ultima Gold) and radioactivity was measured in the scintillation counter, then normalized to the input protein. Before the harvest, the integrity of barrier function was confirmed by measuring trans-epithelial electric resistance with a cell culture volt/ohm-meter (EVOM, World Precision Instruments). Membranes with the tissues were excised from the insets, cut in half and saved for RNA isolation and histology.

### Statistical analysis

Statistical tests are specified in the figure legends. To calculate significances, we used: for normally distributed continuous data (e.g. body weight, plasma triglycerides, qPCR etc.) non-paired two-tailed Student *t*-test with confidence level of 95%; for the discrete data (crypts per organoid) non-parametric one-tailed Mann–Whitney rank test with confidence level of 95%. For the multiple comparisons, we used non-paired one-way ANOVA, with Dunnet post-hoc correction, the alpha 0.05. For the gene expression levels by RNA sequencing, significances are calculated by general linear model with negative binomial distribution; *P* values without correction are shown in the figures. For pathway enrichment analysis, transcripts with log(count per million reads) >1 and *P* < 0.05 were selected and run through MetaCore software pipeline (Thomson Reuters, build 6.21.66768, portal.genego.com). Simple linear regression was used to calculate correlations and Pearson’s *R*^*2*^ coefficient, with confidence interval 95% and two-tailed *P* value aet alpha 0.05. Graphs and statistical analysis were done in GraphPad Prism 8.3.

### Reporting summary

Further information on research design is available in the Nature Research Reporting Summary linked to this article.

## Supplementary information

Supplementary information

Description of Additional Supplementary Files

Supplementary Data 1

Reporting Summary

## Data Availability

Sequencing data associated with this study have been deposited to the Gene Expression Omnibus with accession codes GSE74228, GSE182348, and GSE152056. Source data are provided with this paper. Metabolomic source data are provided in the Supplementary Data 1 of the paper. All other data used in this study are available from the corresponding author upon reasonable request. Source data are provided with this paper.

## Source data

Source Data
