# Supplementary material for: Dietary excess regulates absorption and surface of gut epithelium through intestinal PPARα
Source: Nat Commun. 2021 Dec 2;12:7031. doi: 10.1038/s41467-021-27133-7 (PMC8639731; doi:10.1038/s41467-021-27133-7)
Supplement: Supplementary file 1 — Supplementary information [file 41467_2021_27133_MOESM1_ESM.pdf]

Supplementary Information for

Dietary excess regulates absorption and surface of gut epithelium through intestinal PPARα

Ozren Stojanović, Jordi Altirriba, Dorothée Rigo, Martina Spiljar, Emilien Evrard, Benedek Roska, Salvatore Fabbiano, Nicola Zamboni, Pierre Maechler, Françoise Rohner-Jeanrenaud, Mirko Trajkovski*

* to whom correspondence should be addressed: mirko.trajkovski@unige.ch

The PDF files includes:

Supplementary figures

Supplementary legends

Supplementary tables

Supplementary Figure 1

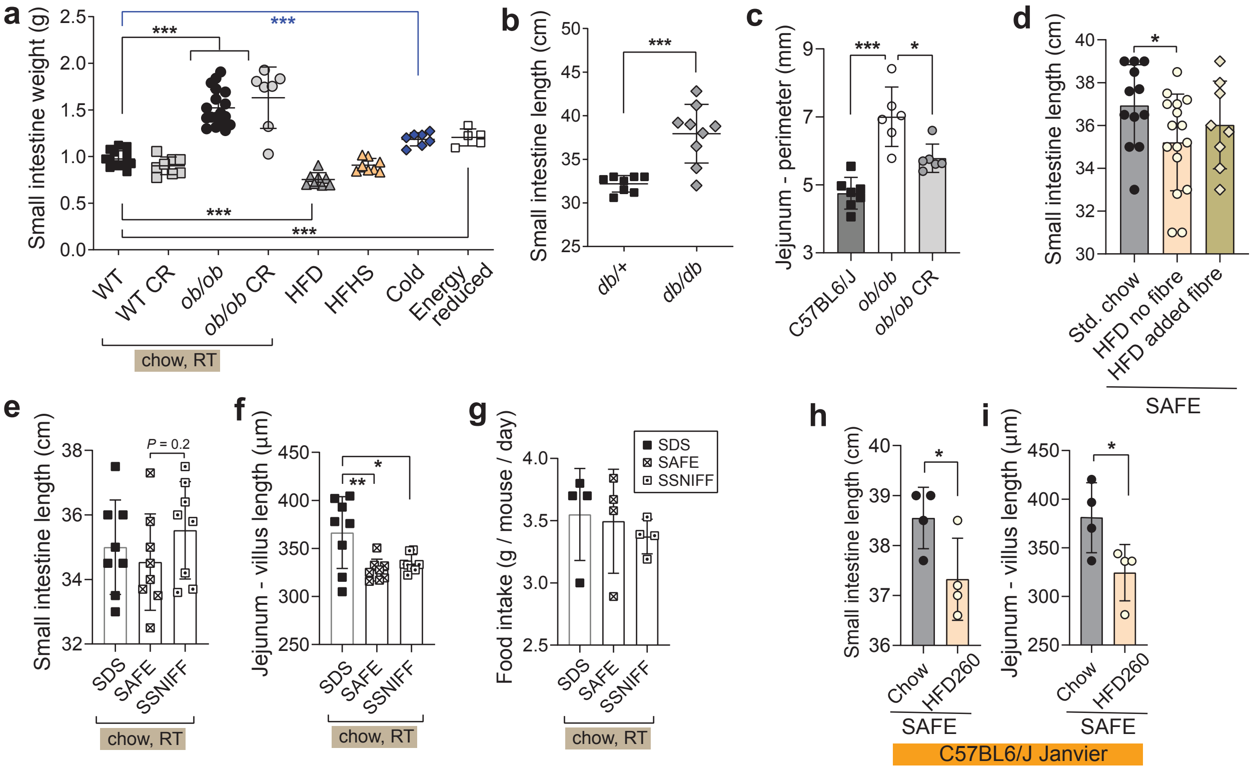

Supplementary Figure 1. Correlation between diet type, food intake and gut size (related to Figure 1). a, Small intestine weight of male mice 14-week-old, C57BL6 background, SPF facility, n=14 (WT), 8 (WT CR), 17 (*ob/ob*), 7 (*ob/ob* CR), 8 (HFD, HFHS), 7 (Cold), 5 (ER) mice.. Abbreviation of the treatments: CR = caloric restriction (60% of ad libitum) for 6 weeks (WT, *ob/ob* groups), HFD = high-fat diet, HF-HS = high-fat high-suvrose diet, RT = room temperature (23°C), Cold = 6°C for 30 days, Energy reduced = *ad libitum* feeding on diet with the low caloric density for 30 days. WT, HFD, HF-HS pooled from two independent experiments. **b,** Small intestine length in 14-week old *db/+* and *db/db* male mice, n=9 per group. **c,** Jejunum perimeter increase in *ob/ob* mice (n=6), and its decrease in *ob/ob* CR (n=6), compared to C57BL6/J WT (n=7). **d,** Small intestine length in C57BL/6J male mice on standard chow or on HFD with no fibre (SAFE HFD260) or with added fibre content (SAFE HFD150), from SAFE (France) for 6 weeks, n=12 mice per group. **e-g**, Comparison of three chow diets in C57BL6/J mice (Charles River France), RD = RM-3 (Special Diets Service), SAFE = Safe 150, SSNIFF = (ssniff), small intestinal length (**e**), villus length (**f**), food intake (**g**), n=8 per group. **h-i**, Intestinal (**h**) and villus length (**i**) in 12-week old C57BL6/J from Janvier fed Safe 150 chow or Safe HFD260 diet (4-week treatment), n=4 mice per group. All data represent mean ± S.D,, * P≤0.05, **P<0.01, ***P<0.001 of unpaired two-sided *t*-test, confidence level 95%. Source data are provided as a Source Data file.

Supplementary Figure 2

Pck1 deletion, check for compensation by the isoform and the key gluconeogenic genes

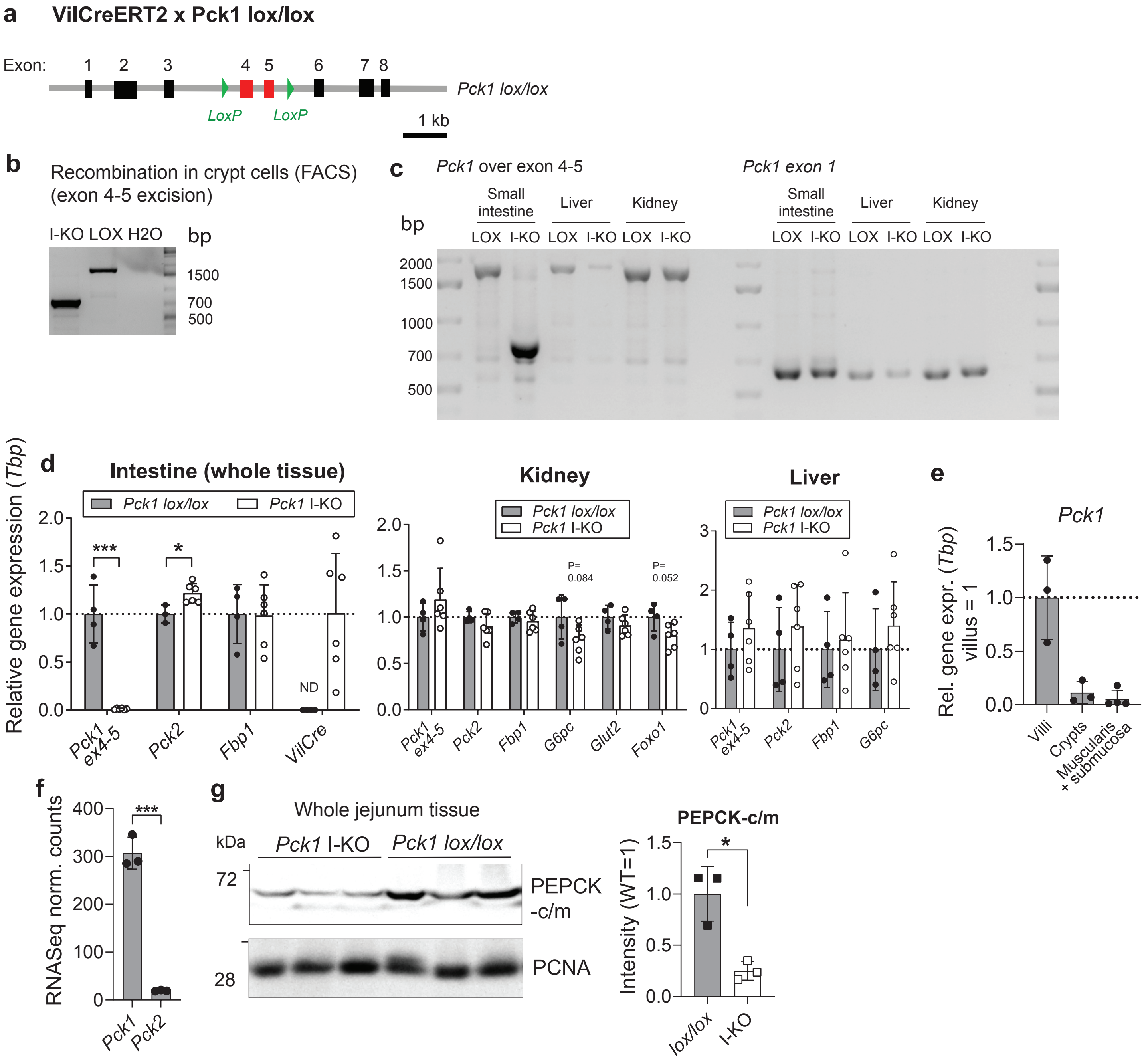

Supplementary Figure 2. Generation of *VilCreERT2* x *Pck1*$^{lox/lox}$ (*Pck1* I-KO) mice. a, targeting exon 4-5 of *Pck1* locus[65]. **b**, PCR over exons 4-5 in sorted epithelial cells from the crypts of *Pck1* I-KO mice injected with tamoxifen. **c**, Agarose gel of PCR over exon 4-5 is small intestine, liver and kidney (*left*), and over exon 1 outside of targeted region (*right*). **d**, Gene expression of Pck1, Pck2 and other gluconeogenic genes by qPCR in jejunum, liver and kidney of Pck1 I-KO (n=4) and lox/lox mice (n=6), after 30 days of cold exposure. **e**, Expression of *Pck1* in villus, crypt and mucosal-serosal domain of jejunum of WT control mice, normalized to *Tbp* and to the levels in the villus, n=4 per group. **f**, RNA levels by RNA sequencing of PCK isoforms in the jejunum of WT control mice, n=3 per group. **g**, WB of proximal jejunum whole tissue for PEPCK and housekeeping gene PCNA, and PEPCK signal quantification (n=3 samples per group). All data represent mean ± S.D,, * P≤0.05, **P<0.01, ***P<0.001 of unpaired two-sided *t*-test confidence level 95%. Source data are provided as a Source Data file.

Supplementary Figure 3

Hk2 recombination, check for compensation by isoform and the key glicolytic genes

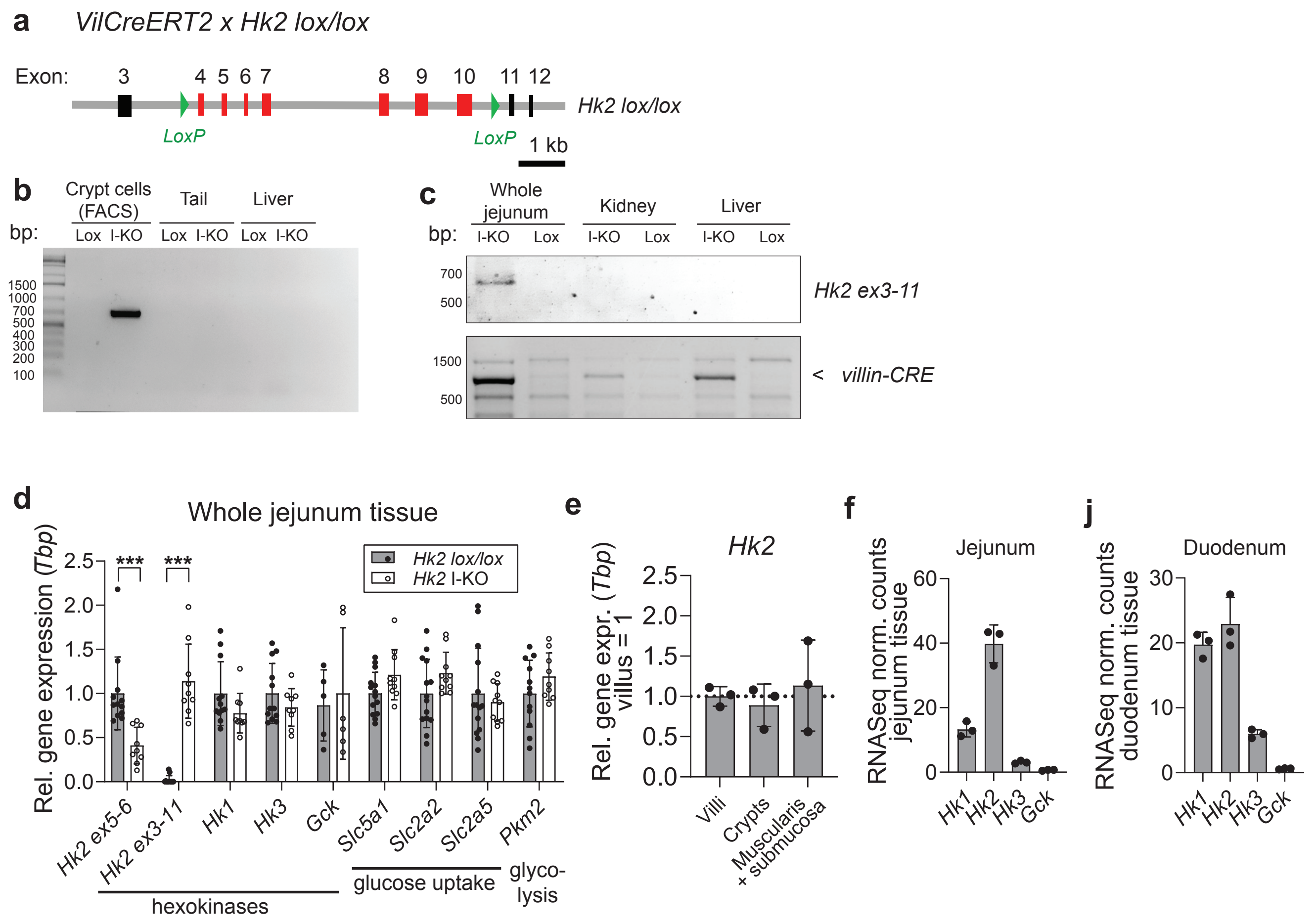

Supplementary Figure 3. Generation of *VilCreERT2* x *Hk2*$^{lox/lox}$ (*Hk2* I-KO) mice. a, targeting exons 4-10 in *Hk2* locus (Hk2$^{tm1.1Uku}$, MGI:5320615 from European Mutant Mouse Archive). **b**, PCR over exons 4-10 in sorted epithelial cells from the crypts of *Hk2* I-KO mice injected with tamoxifen, detectable only upon recombination. **c,** Agarose gel of PCR over Hk2 exons 4-10, and of *villin-Cre* amplification in small intestine, liver and kidney. **d**, Gene expression of Hk2 (excision and recombination), other hexokinase isoforms, glucose uptake and glycolytic genes by qPCR in jejunum of *Hk2* I-KO (n=11) and lox/lox mice (n=10), after 4 months of HFD, two experiments combined. **e**, Expression by qPCR of *Hk2* in villus, crypt and mucosal-serosal domain of jejunum of WT control mice, normalized to *Tbp* and to the levels in the villus, n=4 per group. Note significant expression in non-epithelial fraction, as opposed to other knock-out genes in the study. **f-j**, RNA levels by RNA sequencing of hexokinase isoforms in the jejunum (**f**) and duodenum (**j**) of WT control mice, n=3 per group. All data represent mean ± S.D,, * P≤0.05, **P<0.01, ***P<0.001 of unpaired two-sided *t*-test, confidence level 95%. Source data are provided as a Source Data file.

Supplementary Figure 4

Glud1 deletion, check for compensations

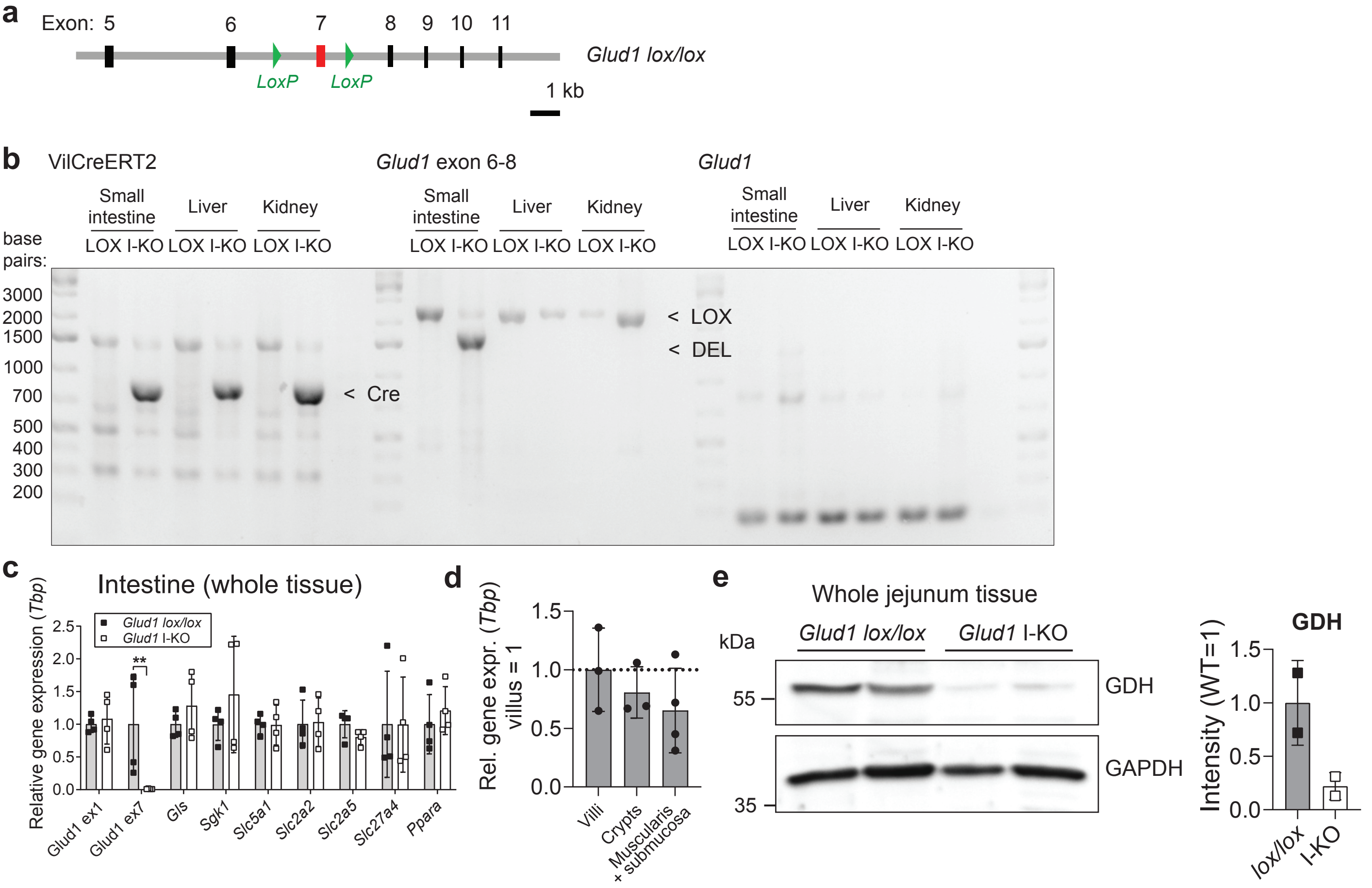

Supplementary Figure 4. Generation of *VilCreERT2 x Glud1$^{lox/lox}$* (*Glud1* I-KO) mice. **a**, Targeting exon 7 on Glud1 locus. **b**, PCR of *villin Cre*, *Glud1* over excised exon 7 and non-excised part in small intestine, liver and kidney. **c**, Gene expression of *Glud1* (non-excised and excised part), glutaminase, the genes of carbohydrate uptake, and of *Ppara*, by qPCR in jejuna of *Glud1 lox/lox* and I-KO and mice, after 4 months of HFD, from one experiment (n=4 per group). **d**, Expression by qPCR of *Glud1* in villus, crypt and mucosal-serosal domain of jejunum of WT control mice, normalized to *Tbp* and to the levels in the villus, n=4 per group. **e**, WB of proximal jejunum whole tissue for glutamate dehydrogenase (GDH) and housekeeping protein GAPDH, and GDH signal quantification (n=2 per group). All data represent mean ± S.D,, * P≤0.05, **P<0.01, ***P<0.001 of unpaired two-sided *t*-test, confidence level 95%. Source data are provided as a Source Data file.

Supplementary Figure 5

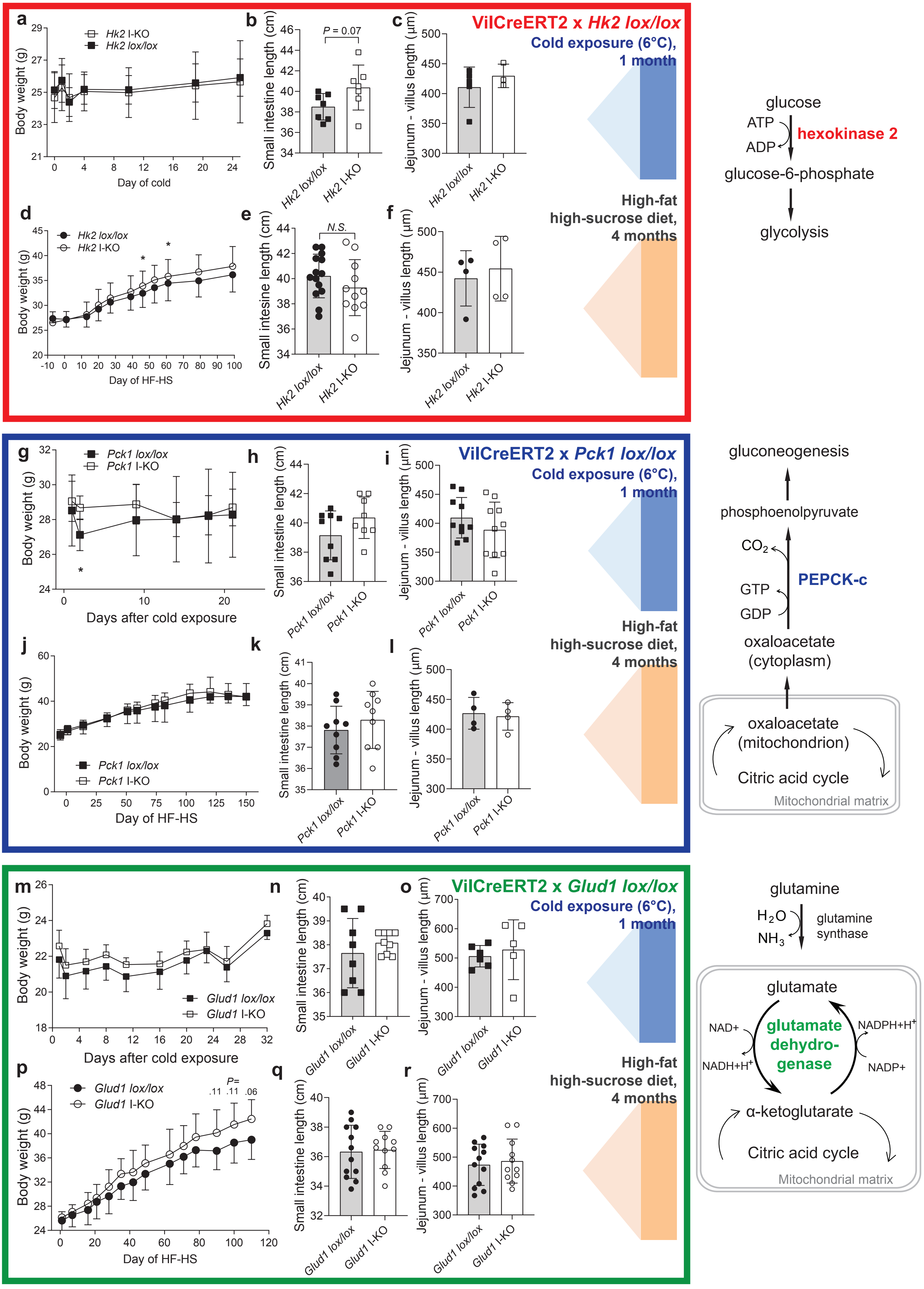

Supplementary Figure 5. Intestinal deletion of rate-limiting enzymes for glycolysis, gluconeogenesis and glutamate dehydrogenase does not impair intestinal adaptive expansion on cold, and does not reduce BW gain on high-fat high-sucrose diet (HF-HS). The treatments started at age 8-9 weeks, one week after tamoxifen induction and lasted as indicated. All mice were male, and SPF housed. Body weight, small intestinal length and average villi length in jejunum shown for every experiment, left to right. **a-f,** *VilCreERT2 x Hk2* male mice on cold and HF-HS, n=7 per group (**a, b**), n=4 (lox/lox) and 3 (I-KO) mice per group (**c**), n=11 per group (**d, e**), n=4 per group (**f**). **g-l,** *VilCreERT2 x Pck1* male mice on cold and HF-HS, n=9 (lox/lox) and 8 (I-KO) mice per group (**g-h**), n=10 per group (**i**), n=9 per group (**j-k**) and n=4 per group (**l**). **m-r,** *VilCreERT2 x Glud1* male mice on cold and HF-HS, n=8 (lox/lox), 9 (I-KO) mice (**m-n**), n=6 (lox/lox), 5 (I-KO) mice (**o**), n=12 (lox/lox), 11 (I-KO) mice (**p-r**). The graphs are pools of two independent experiments, except **c**, **f**, **l**, and **o**, which are from one experiment. All data represent mean ± S.D, * P≤0.05, **P<0.01, ***P<0.001 of unpaired two-sided *t*-test, confidence level 95%.

Supplementary Figure 6

Genotyping of *Ppara* I-KO and *Ppara lox/lox* mice, check for isoform compensation

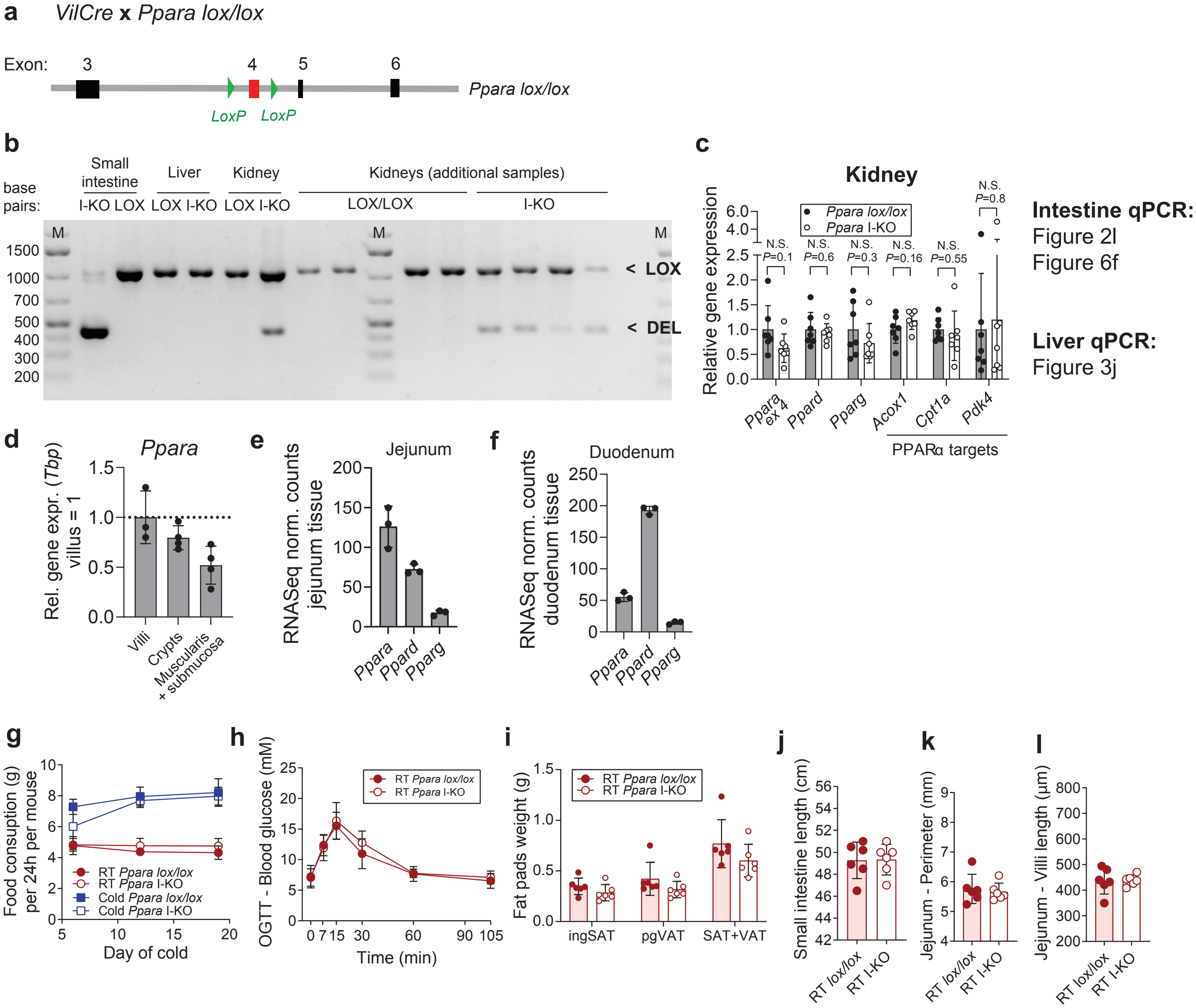

Supplementary Figure 6. Generation of *Ppara* I-KO mice. a, Targeting exon 4 of *Ppara* gene. **b**, Agarose gel of the PCR amplification over exon 4, recombination in intestine, and partial leakage in kidney. **c**, expression of PPAR genes by qPCR in kidneys of *Ppara* I-KO and *lox/lox* mice, n=7 per group. **d**, Expression by qPCR of *Ppara* in villus, crypt and serosal domain of jejunum of WT control mice, normalized to *Tbp* and to the levels in the villus, n=4 per group. **e-f**, RNA levels by RNA sequencing of PPAR isoforms in the jejunum (**e**) and duodenum (**f**) of WT control mice, n=3 per group. **g-l,** Daily food consumption (**g**), oral glucose tolerance test (**h**), fat pad weights (ingSAT = inguinal subcutaneous, pgVAT = perigonadal visceral adipose tissue) (**i**), small intestine length (**j**), average villi length in proximal jejuna (**k**) and perimeter of proximal jejuna (**l**) in the room temperature (RT) *Ppara lox/lox* and *Ppara* I-KO male mice, 16 week-old, n=6 per group, RT controls for the cold-exposed mice in the main Fig. 2f-j. All data represent mean ± S.D, * $P \leq 0.05$, **$P<0.01$, ***$P<0.001$ of unpaired two-sided *t*-test, confidence level 95%. Source data are provided as a Source Data file.

Supplementary Figure 7

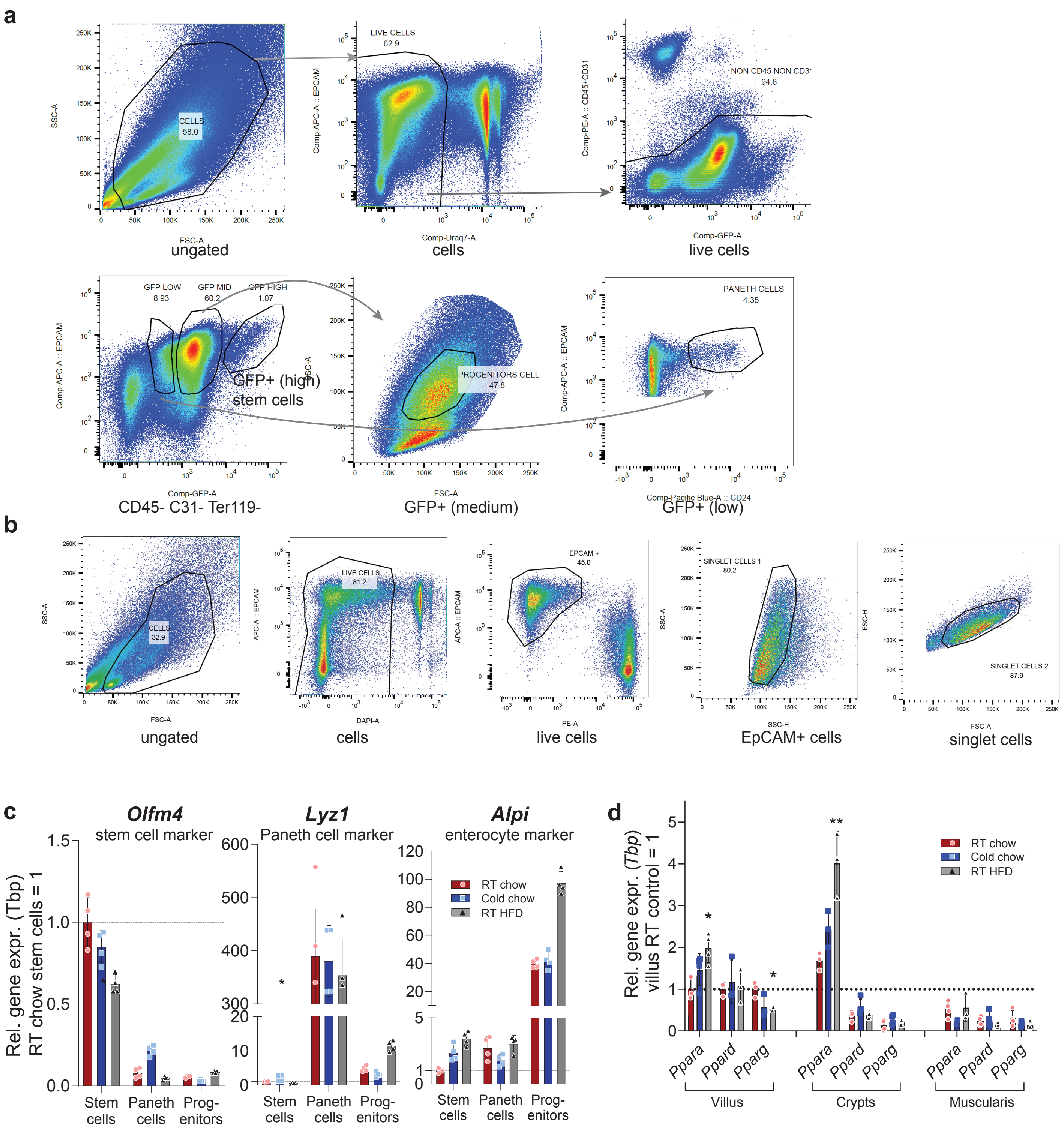

Supplementary Figure 7. Sorting of epithelial cells from Lgr5-GFP mice exposed two weeks to cold (6°C) or HFD (related to Figure 2n)**. a,** Gating strategy for FACS of the crypt cells into stem, Paneth, and transient progenitor population. Blood cells marked by CD45, CD31 and TER119 were excluded, stem cells were selected according to strong GFP signal, Paneth cells were marked by high CD24 and low GFP signal, and progenitor cells by low CD24, low GFP, and EpCAM+. **b**, FACS of epithelial cells from villus domain (EpCAM+, all subtypes). **c**, Purity and specificity of FACS was controlled by qPCR of marker genes for the sorted types, n=4 per group (2 replicate isolation from 2 mice). **d**, Villus, crypt and mucosal-serosal fraction of jejunum were separated, RNA isolated, and gene expression analysed by qPCR, normalization to *Tbp* and villus level, n=4 mice per group. All data represent mean ± S.D, * $P \leq 0.05$, **$P<0.01$, ***$P<0.001$ of unpaired two-sided *t*-test, confidence level 95%. Source data are provided as a Source Data file.

Supplementary Figure 8

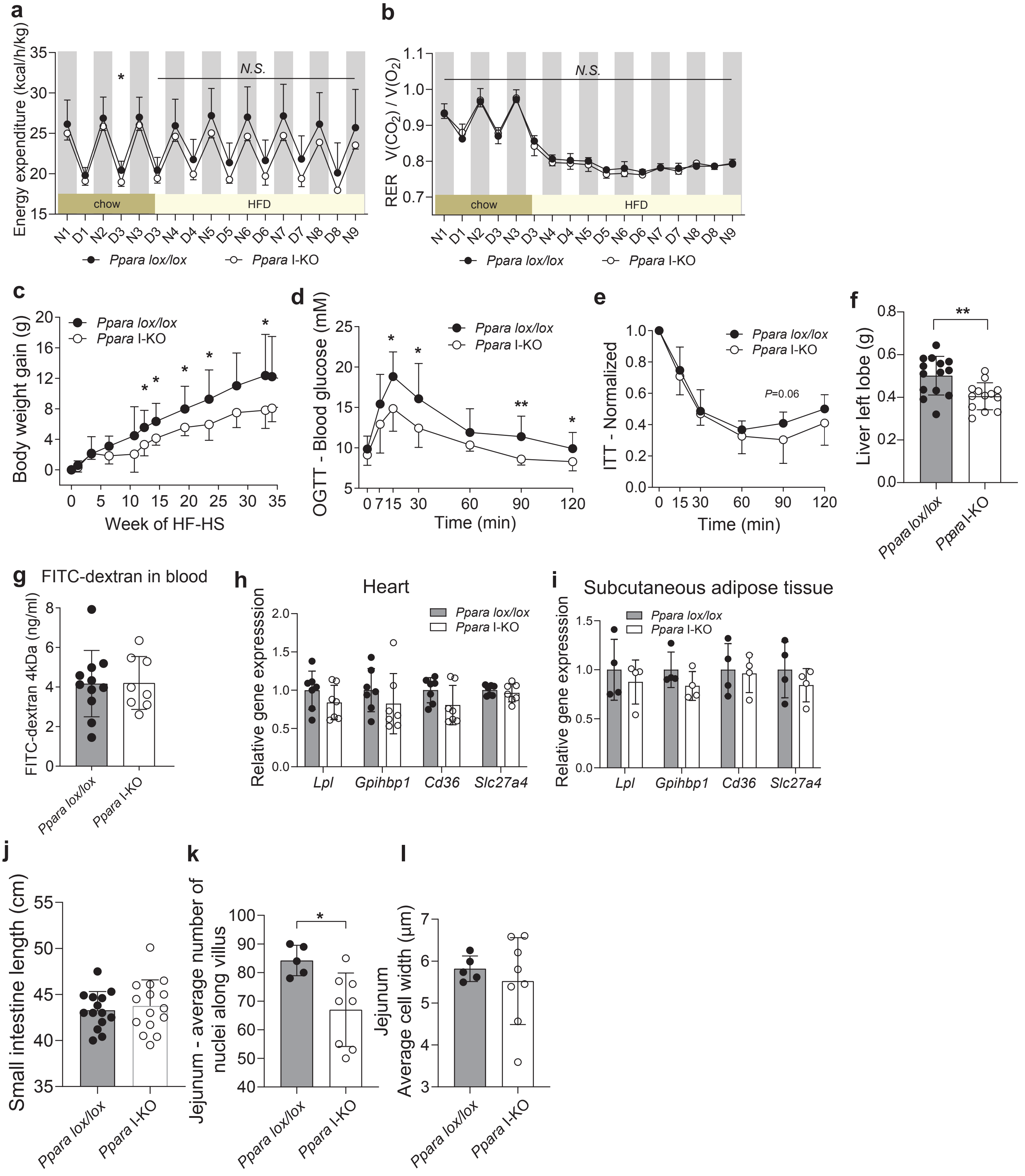

Supplementary Figure 8. *Ppara* I-KO reduces weight gain and improves oral glucose tolerance during HFD and HF-HS diet. **a-b,** Energy expenditure normalized to lean mass (**a**) and respiratory quotient V(CO_2) / V(O_2) (**b**), measured in the metabolic cage, of *Ppara lox/lox* and I-KO mice on HFD, n=5 per group. Shaded periods are 12h dark phases. **c-e,** Body weight gain (**c**), OGTT (**d**) and ITT (**e**) in 6h fasted mice of mice on HF-HS diet (from the age of 8 weeks), n=7 for *Ppara lox/lox*, n=9 for *Ppara* I-KO. **f,** Liver left lobe weight of HFD mice, n=14 per group, pooled from two experiments. **g**, Concentration of FITC-dextran 4kDa in plasma, 4 hours after oral gavage, in *Ppara lox/lox* and *Ppara* I-KO mice on HF-HS diet (*Ppara lox/lox* n=11, *Ppara* I-KO n=8). **h-i,** Relative qPCR gene expression normalized to *Tbp* in heart (**h**) and inguinal SAT (**i**) in 6h fasted HFD mice, n=8 per group. **j,** Small intestine length on HFD, n=12 per group. **k-l,** Average number of nuclei (counted on H&E sections) along one side of villus in jejunum (**k**) and average epithelial cell width along the villus (**l**), on HFD, from n=5 (lox/lox), 8 (I-KO) mice per group. All data represent mean ± S.D,, * P≤0.05, **P<0.01, ***P<0.001 of unpaired two-sided *t*-test, * P≤0.05, **P<0.01, ***P<0.001 of *t*-test. Source data are provided as a Source Data file.

Supplementary Figure 9

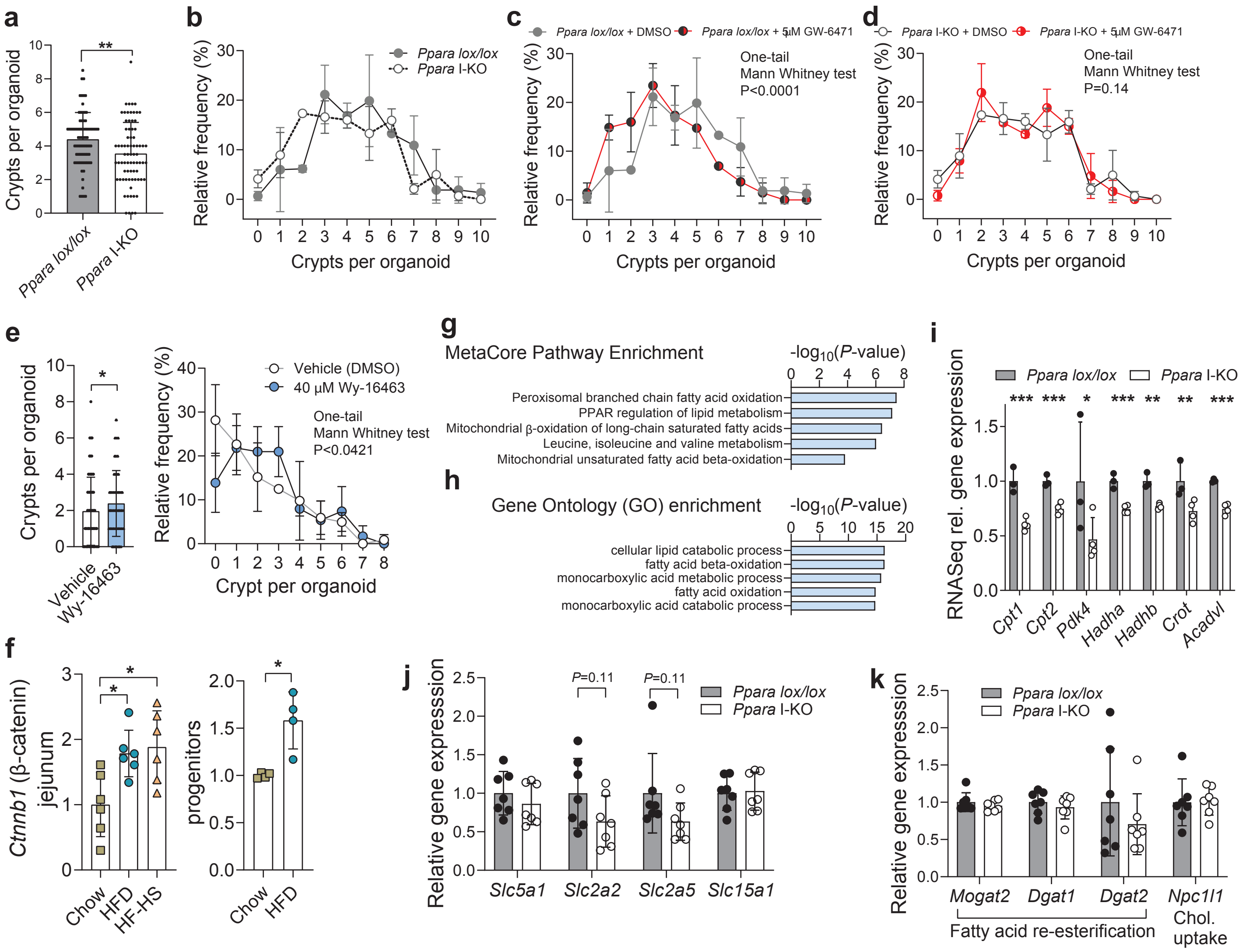

Supplementary Figure 9. Supplemental characterisation of the *Ppara* I-KO intestines and crypts. a-b, Number of crypts per organoid (**a**) and histogram of the organoids **(b)**, derived from two *Ppara lox/lox* and I-KO mice on HFD for 2 months, cultured without inhibitors, counted on day 7. N=53 (*lox/lox*) and 75 (I-KO) organoids (**a**) analysed in N=2 experiments (**b**). **c-d,** Histogram of *Ppara lox/lox* (**c**) and *Ppara* I-KO organoids (**d**), from HFD mice, with and without PPARα inhibitor, counted at day 7. **a-d** are pooled from n=2 mice per group. **e**, Histogram of WT organoids from HFD mouse, treated daily with PPARα agonist Wy-16463, n=3 wells per group. Mann-Whitney tests on the histograms refer to comparison of all organoids between the two groups. **f,** β-catenin mRNA levels (qPCR, normalized to *Tbp*) in proximal jejuna (left, n=6 per group) or sorted progenitors from jejuna (right, n=4 per group) of WT mice fed chow or HFD diets. **g-i,** MetaCore pathway enrichment analysis (**g**), GO term enrichment (**h**), and relative expression of the key fatty acid oxidation genes (**i**) analysed from RNA sequencing of whole tissue duodena of *Ppara lox/lox* (n=3 mice) and *Ppara* I-KO (n=4 mice), following 8-week HFD. **j-k,** Relative qPCR expression of genes for glucose and peptide uptake (**j**) and fatty acid re-esterification (**k**) in jejuna from mice on HFD for 8 weeks, n=7 per group, normalized to *Epcam* (epithelial marker). All data represent mean ± S.D, * $P \leq 0.05$, **$P<0.01$, ***$P<0.001$ of unpaired two-sided *t*-test, confidence level 95%, except for **a** where is for one-tailed Mann-Whitney non-parametric test, and **i** general linear model with negative binomial distribution, without correction for multiple compaarison (for RNASeq). Source data are provided as a Source Data file.

Supplementary Figure 10

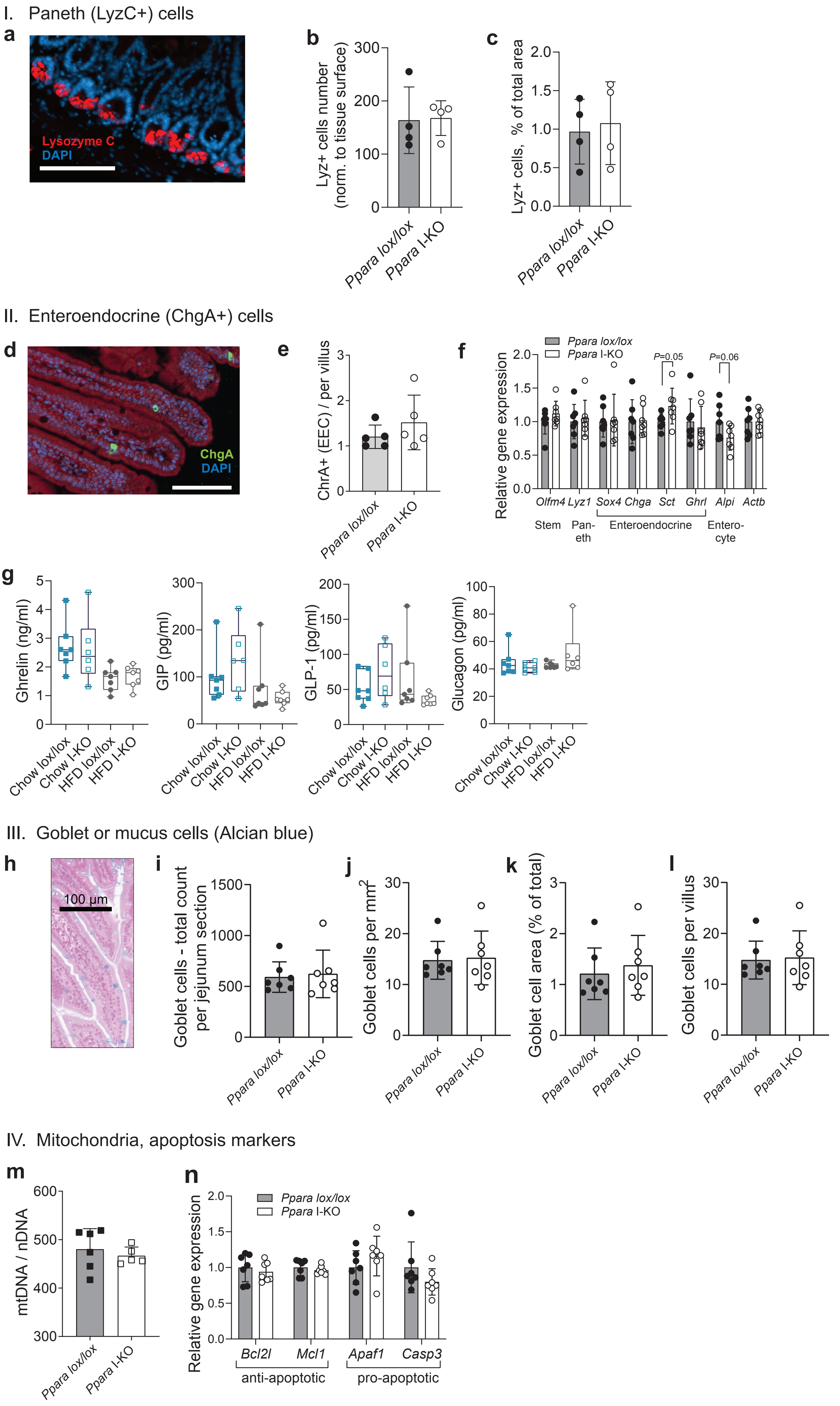

Supplementary Figure 10. Intestinal cell-type characterization in *Ppara* I-KO jejunua on HFD. a-c, Staining against Paneth cell marker lysozyme C (**a**), number of positive cells normalized to tissue surface (**b**), and their surface area as a percentage of total tissue area (**c**), n=5 per group. **d-e,** Staining against endocrine marker chromogranin A (**d**), average number of ChgA+ cells per villus, quantified in proximal jejuna of n=5 (*lox/lox*) and 4 (I-KO) mice. (**e**). **f**, Relative qPCR expression of cell markers, including EEC in proximal jejuna following 8-week HFD, n=7 per group, normalized to *Tbp*. **g,** Concentration of major incretins in fasted plasma of mice on standard chow or HFD, n=7 per group (HFD I-KO=6). Box represents 25th to 75th percentile, whiskers min to max, central bar is mean. **h-l**, staining for mucus cells by Alcian blue (**h**), and quantification of number of positive cells per section (**i**), per surface area (**j**), by cell area as a percentage to total tissue area (**k**), and cell number per villus (**l**), n=7 per group. **m,** Mitochondrial content in proximal jejuna of HFD mice measured by qPCR of mitochondrial and nuclear DNA, expressed as a ratio of *mt-Nd1* and *Tbp*, n=5 per group. **n**, Relative qPCR of pro- and anti-apoptotic genes in the same samples as in Supplementary Figure 9j-k. Scale bars represent 100 µm. All data represent mean ± S.D, * $P \leq 0.05$, **$P<0.01$, ***$P<0.001$ of unpaired two-sided *t*-test, confidence level 95%, unless stated otherwise. Source data are provided as a Source Data file.

Supplementary Figure 11

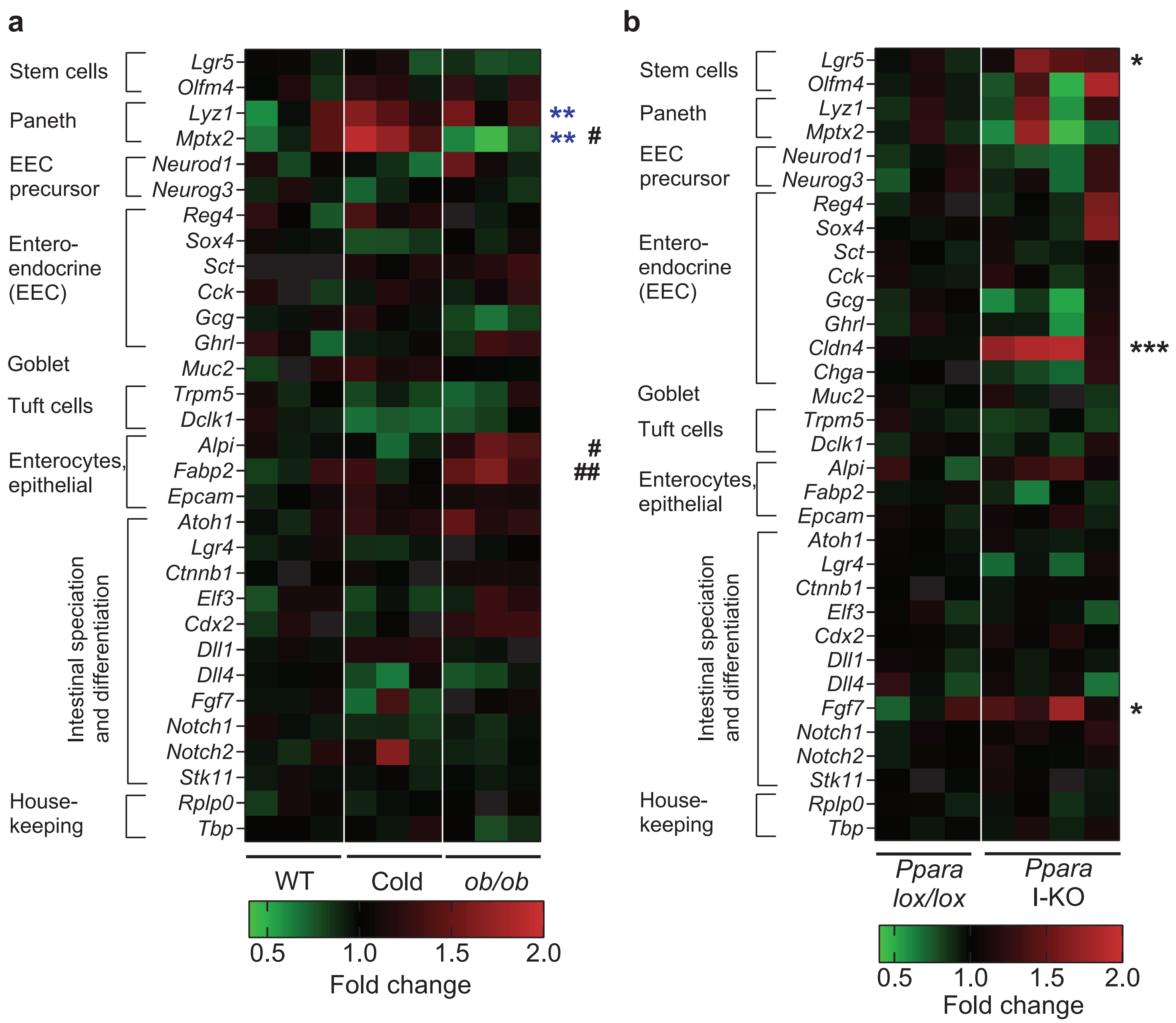

Supplementary Figure 11. Markers of cell types, intestinal division and differentiation, by RNA sequencing in hyperphagic, and in *Ppara* I-KO mice. a, Gene expression in whole jejunum tissue of WT mice on RT or after 30-day cold-exposuere, and in ob/ob mice fed ad libitum on RT, from the same dataset as in Figure 2 a, b, d. * marks significance between WT and Cold, # between WT and *ob/ob*. **b,** Gene expression in whole duodenum tissue of *Ppara lox/lox* and I-KO on HFD, from the same dataset as in Supplementary Figure 9g-i. * or # $P \leq 0.05$, ** or ## $P<0.01$, *** or ### $P<0.001$ of general linear model with negative binomial distribution. Source data are provided as a Source Data file.

Supplementary Table 1. List of primers used in qPCR experiments

MOUSE

Gene	Forward primer (5' > 3')	Reverse primer (5' > 3')
Acox1	tcgaagccagcgttacgag	atctccgtctgggcgtagg
Adfp (Plin2)	cactccactgtccacctgatt	tcctgagcaccctgaatttt
Apoa4	cacacagacccaggaaatga	ccttgatcgtggtctgcat
Cd36	ttgaaaagtctcggacattgag	tcagatccgaacacagcgta
Cpt1a	gactccgctcgctcattc	tctgccatcttgagtggtga
Ctnnb1	tgcagatcttggactggacat	aagaacggtagctgggatca
Dgat1	tttcagcaattatcgtggtatcc	aaaaataaccttgcattactcagga
Dgat2	gctggtgccctactccaag	ccagcttggggacagtga
Epcam	tgtcatttgctccaaactgg	gttctggatcgccccttc
Fasn	cctggatagcattccgaacct	agcacatctcgaaggctacaca
Gk	tccaggaaataataactttgtcaagtc	cactgcactgaaatacgtgct
Gpihbp1	gctgcaatcagacacagagc	gtaggtagtcaggtaacctttgtcg
Hmgcs2	ataccaccaacgcctgttatgg	caatgtcaccacagaccaccag
Lpl	tttgtgaaatgccatgacaag	cagatgctttcttctcttgtttgt
M6prbp1 (Plin3)	aggacccagctaagccagag	agggccacacacatgctc
Mogat2	tacagctttggcctcatgc	agggctgtggtgtcatctg
Npc1L1	ctttgtggccctgctctc	gcttgaaaagcagcacacg
Pdk4	cgcttagtgaacactccttcg	cttctgggctcttctcatgg
Ppara	agagccccatctgtcctctc	actggtagtctgcaaaaccaaa
Ppard	tgcagatgggctgtgatggg	ctcgagcttcatgcggattgtc
Pparg	aggcgagggcgatcttgacag	aattcggatggccacctctttg
Plin2	cactccactgtccacctgatt	tcctgagcaccctgaatttt
Plin3	aggacccagctaagccagag	agggccacacacatgctc
Prdm16	cagcacggtgaagccatt	gcgtgcatccgcttgtg
Rxra	acatgcagatggacaagacg	gggtttgagagccccttaga
Slc15a1	agacaagctgacaagcataaaca	catgagctactgtggtgactcc
Slc27a2	atctagcggaaagcctctgg	tgccacagccaactcaga
Slc27a4	cttgcctgagctgcacaa	gcgggtctttcacaacagat
Slc2a2	gtcagctattcatccacattcagt	agccaaggttccggtgat
Slc2a5	agagcaacgatggaggaaaa	ccagagcaaggaccaatgtc
Slc5a1	ctggcaggccgaagtatg	ttccaatgttactggcaaagag
Tbp	gaagctgcggtacaattccag	ccccttgtacccttcaccaat

HUMAN

Gene	Forward primer (5' > 3')	Reverse primer (5' > 3')
ACOX1	gaagtggtgtagaacccttcca	ccccagggtaagtctggtaga
ADFP (PLIN2)	actggctggtaggtccctttt	caccttggtcctgagcattc
CD36	tcccaagctcaagtgaatctc	atgccagttgaatgcctacc
PLIN3	tgagcttctgagcctcattg	catttcaacaaggcttaccaca
PDK4	cagaccagttgcgctgatta	tgcttccctctcttcctatatcc
PPARA	gctgtgtgcacctccctaat	gtctgaagatgggcttgaatg
SLC15A1	tgcaggtggaaatcgataaa	aactttaatttggacttcgtttcc
SLC27A4	ccgctacctcctgaaccag	ctagtgccatgcgaacctg
SLC2A2	ccctgtctgtatccagctttg	tgtttgctactaacatggctttg

Supplementary Table 2. Mouse food used in the study (based on manufacturers information)

significant differences

HFD
ssniff, D12492 (E15742-34)
HF diet for rodents

Source: lard & soybean oil

**Gross Energy**	**25.0 MJ/kg**
**Metabolizable Energy**	**21.6 MJ/kg**

**Calories**	
Carbohydrates	20.0
Protein	20.0
Fat	60.0

**Crude Nutrients [%]**	
Crude protein (N x 6.25)	24.4
Crude fat	34.6
Crude fibre	6.0
Crude ash	5.3
Starch	0.1
Sugar	9.4
N free extracts	26.3

**Fatty acids [%]**	
C 12:0	0.07
C 14:0	0.44
C 16:0	7.93
C 18:0	4.37
C 20:0	0.11
C 16:1	0.94
C 18:1	13.97
C 18:2	4.64
C 18:3	0.49

**Dietary composition %**	
Casein	27.700
Corn starch	—
Maltodextrin	15.600
Sucrose	8.460
Cellulose powder	6.000
L-Cystine	0.350
Vitamin premix	1.000
Mineral & trace element premix	6.000
Choline chloride (50 % choline)	0.250
Dye (blue)	0.030
Butylated hydroxytoluene	0.010
Pork lard	31.500
Soybean oil	3.100

HF-HS
ssniff, D12331 (EF D12331 mod)
Surwit with sucrose

Source: hydrogenated coconut oil

**Gross Energy**	**25.1 MJ/kg**
**Metabolizable Energy**	**22.6 MJ/kg**

**Calories [%]**	
Carbohydrates	25.0
Protein	15.0
Fat	59.0

**Crude Nutrients [%]**	
Crude protein (N x 6.25)	20.2
Crude fat	35.7
Crude fibre	0.5
Crude ash	5.1
Starch	—
Sugar	17.9
N free extracts	34.1

**Fatty acids [%]**	
C 6:0	0.10
C 8:0	1.97
C 10:0	1.60
C 12:0	15.22
C 14:0	6.54
C 16:0	3.80
C 18:0	4.26
C 20:0	0.06
C 16:1	0.01
C 18:1	0.74
C 18:2	1.23
C 18:3	0.14

**Dietary composition %**	
Casein	23.000
Corn starch	—
Maltodextrin	15.300
Sucrose	17.000
Cellulose powder	1.800
L-Cystine	0.200
Vitamin premix *	1.000
Mineral & trace element premix	5.800
Choline chloride	0.200
Dye (yellow & red)	0.100
Coconut oil, hydrogenated	33.300
Soybean oil	2.300

**contains sucrose*

HFD260 (“no fiber”)
SAFE, HF260 (U8978 Version 19)

Source: butter-based

Metabolizable Energy (Atwater) **23.1 MJ/kg**

**Calories**	**[%]**
Carbohydrates	26.8
Protein	14.4
Fat	58.6

**Crude Nutrients [%]**	
Crude protein (N x 6.25)	20.0
Crude fat	36.0
(All carbohydrates)	36.7
Starch	14.5
Sugars	20.2
Sucrose	17.9
Cellulose	0.0
Minerals	4.2

**Fatty acids [%]**	
Saturated	23.31
Unsaturated	9.4
Monounsaturated	7.1
Polyunsaturated	2.7

HFD150 (“added fiber”)
SAFE (modified HFD260)

Source: butter-based

Metabolizable Energy (Atwater) **22.2 MJ/kg**

Cellulose	1.4

Energy-reduced food
ssniff (V9631-S710)

**Gross Energy (GE)**	**14.6 MJ/kg**
**Metabolizable Energy (ME)**	**7.7 MJ/kg**

**Calories**	**[%]**
Carbohydrates	11.0
Protein	51.0
Fat	11.0

**Crude Nutrients [%]**	
Crude protein (N x 6.25)	17.5
Crude fat	2.6
Crude fibre	17.3
NDF	31.2
ADF	17.7
Crude ash	7.5
Starch	9.5
Sugar	4.8
N free extracts	36.4

The energy density of this special diet has been reduced by 30 – 40 % when compared with a conventional maintenance or a breeding diet, respectively.

CHOW ssniff
(ssniff, Germany)
V1554-703

Source: vegetal diet, no soybean, alfalfa, low phytoestrogens

**Gross Energy**	**14.74 MJ/kg**
**Metabolizable Energy**	**13.75 MJ/kg**

**Calories**	**[%]**
Carbohydrates	67.0
Protein	23.0
Fat	10.0

**Crude Nutrients**	**[% weight]**
Crude protein (N x 6.25)	19.1
Crude fat	3.4
Crude fibre	4.6
NDF	20.6
ADF	6.5
Crude ash	6.0
Starch	34.5
Sugar	3.3
N free extracts	55.1

Dietary composition:
Wheat, barley, corn gluten, potato proteins, minerals, oat hulls, vitamins and trace elements vegetable oil, L-lysine HCl, DL-methionine

CHOW SAFE
(SAFE Diets, France)
SAFE-150

Source: vegetal diet, no soybean, alfalfa, low phytoestrogens

**Gross Energy**	**16.4 MJ/kg**
**Metabolizable Energy**	**13.7 MJ/kg**

**Calories**	
Carbohydrates	66.4
Protein	21.0
Fat	12.6

**Crude Nutrients**	**[% weight]**
Nitrogen Free Extract	56.9 %
of which Starch	41.0 %
of which Sugars	3.4 %
Crude Protein	18.0 %
Crude Fat	4.8 %
Crude Ash	4.2 %
Crude Fiber	4.1 %
Moisture	12.0 %

Dietary composition:
Barley, wheat, maize, maize gluten, wheat germ, wheat bran, potato protein, sunflower seed, inactivated brewer's yeast, calcium carbonate, pre-mixture of vitamins, pre-mixture of minerals, dicalcium phosphate, L-lysine, DL-methioni

Chow SDS
(Special Diet Services)
Rat and Mouse No.3 Breeding (RM3)
RM3 (E) SQC (811181)

Source: soya, fish-meal, wheat (see below)

**Gross Energy**	**15.21 MJ/kg**
**Metabolizable Energy**	**13.9 MJ/kg**

**Calories**	**[%]**
Carbohydrates	61.57
Proteins	26.93
Fat	11.5

**Crude Nutrients**	**[% weight]**
Glucides	51.59
Proteins	22.39
Fibers	4.21
Ash minerals	7.56
Lipids	4.25
Humidity	10

Dietary composition:
Wheat, Wheatfeed, De-hulled Extracted Toasted Soya, Barley, Fish Meal, Whey Powder, Macro Minerals, Yeast, Soya Oil, Vitamins, Micro Minerals, Amino Acids.

Supplementary Table 3: Enrichment of GO processes in RNA sequencing data from Ppara I-KO vs. Ppara lox/lox mice on HFD diet, from duodenum tissue.

Enrichment analysis report					
Enrichment by GO Processes					
#	Processes	Total	pValue	Min FDR	
1	lipid metabolic process	1643	4.768E-22	3.068E-18	
2	cellular lipid metabolic process	1277	1.578E-21	5.077E-18	
3	monocarboxylic acid metabolic process	717	2.778E-21	5.959E-18	
4	cellular lipid catabolic process	281	4.647E-20	7.477E-17	
5	lipid catabolic process	407	1.953E-19	2.514E-16	
6	fatty acid metabolic process	449	8.022E-19	8.605E-16	
7	small molecule metabolic process	2290	1.577E-18	1.450E-15	
8	monocarboxylic acid catabolic process	148	3.460E-18	2.784E-15	
9	fatty acid catabolic process	118	3.947E-18	2.823E-15	
10	carboxylic acid metabolic process	1160	6.741E-18	4.338E-15	
11	response to organic substance	4903	2.019E-17	1.050E-14	
12	carboxylic acid catabolic process	320	2.122E-17	1.050E-14	
13	organic acid catabolic process	320	2.122E-17	1.050E-14	
14	organic acid metabolic process	1306	2.998E-17	1.378E-14	
15	oxoacid metabolic process	1273	3.442E-17	1.477E-14	
16	response to drug	2022	4.144E-16	1.667E-13	
17	regulation of lipid metabolic process	680	1.436E-15	5.437E-13	
18	lipid oxidation	106	1.689E-15	6.038E-13	
19	small molecule catabolic process	541	2.573E-15	8.716E-13	
20	fatty acid beta-oxidation	68	3.101E-15	9.980E-13	
21	fatty acid oxidation	100	8.740E-15	2.679E-12	
22	response to oxygen-containing compound	2993	1.949E-14	5.701E-12	
23	cellular response to chemical stimulus	4593	3.854E-14	1.078E-11	
24	oxidation-reduction process	1285	4.074E-14	1.092E-11	
25	vascular process in circulatory system	351	1.027E-13	2.643E-11	
26	response to organic cyclic compound	1979	1.217E-13	3.014E-11	
27	regulation of biological quality	5819	1.276E-13	3.042E-11	
28	cellular response to toxic substance	385	1.759E-13	4.043E-11	
29	response to lipid	1847	3.430E-13	7.612E-11	
30	response to chemical	7112	3.721E-13	7.984E-11	
31	negative regulation of multicellular organismal process	1914	6.279E-13	1.304E-10	
32	regulation of anatomical structure size	841	2.056E-12	4.135E-10	
33	response to hormone	1876	6.116E-12	1.193E-09	
34	response to toxic substance	1058	6.941E-12	1.314E-09	
35	metabolic process	10952	1.246E-11	2.292E-09	
36	response to endogenous stimulus	2742	1.308E-11	2.338E-09	
37	regulation of tube size	290	1.408E-11	2.449E-09	
38	regulation of locomotion	1448	1.786E-11	3.025E-09	
39	regulation of cell migration	1293	2.293E-11	3.784E-09	

40	regulation of localization	4092	2.356E-11	3.790E-09	
41	cellular response to oxygen-containing compound	2030	2.731E-11	4.288E-09	
42	substantia nigra development	86	3.576E-11	5.480E-09	
43	response to acid chemical	883	3.750E-11	5.612E-09	
44	rhythmic process	546	5.345E-11	7.818E-09	
45	cellular metabolic process	9840	5.706E-11	8.160E-09	
46	cellular response to organic substance	3784	5.946E-11	8.319E-09	
47	regulation of cell motility	1377	7.556E-11	1.000E-08	
48	regulation of blood vessel diameter	288	7.752E-11	1.000E-08	
49	regulation of tube diameter	288	7.752E-11	1.000E-08	
50	regulation of cellular component movement	1504	7.770E-11	1.000E-08	
51	long-chain fatty acid transport	92	9.158E-11	1.154E-08	
52	postsynapse organization	192	9.326E-11	1.154E-08	
53	response to antibiotic	742	1.612E-10	1.957E-08	
54	positive regulation of cell migration	817	1.785E-10	2.127E-08	
55	cellular response to antibiotic	280	2.754E-10	3.223E-08	
56	circulatory system process	722	2.901E-10	3.334E-08	
57	terpenoid metabolic process	182	2.957E-10	3.339E-08	
58	regulation of wound healing	256	3.207E-10	3.559E-08	
59	response to organonitrogen compound	1982	4.882E-10	5.326E-08	
60	neural nucleus development	124	5.465E-10	5.862E-08	
61	regulation of hormone levels	929	6.172E-10	6.507E-08	
62	cellular catabolic process	2370	6.301E-10	6.507E-08	
63	positive regulation of cell motility	854	6.370E-10	6.507E-08	
64	regulation of fatty acid metabolic process	168	6.498E-10	6.535E-08	
65	diterpenoid metabolic process	170	7.814E-10	7.737E-08	
66	regulation of system process	1018	8.695E-10	8.479E-08	
67	cellular response to drug	791	9.424E-10	9.053E-08	
68	lipid modification	275	1.184E-09	1.121E-07	
69	response to steroid hormone	765	1.374E-09	1.254E-07	
70	central nervous system development	1713	1.377E-09	1.254E-07	
71	regulation of response to wounding	305	1.384E-09	1.254E-07	
72	organic substance metabolic process	10219	1.422E-09	1.269E-07	
73	positive regulation of locomotion	879	1.440E-09	1.269E-07	
74	fatty acid transport	113	1.503E-09	1.307E-07	
75	regulation of focal adhesion assembly	95	1.676E-09	1.420E-07	
76	regulation of cell-substrate junction assembly	95	1.676E-09	1.420E-07	
77	catabolic process	2718	1.705E-09	1.425E-07	
78	positive regulation of cellular component movement	890	2.040E-09	1.663E-07	
79	isoprenoid metabolic process	205	2.041E-09	1.663E-07	
80	system development	6693	2.129E-09	1.698E-07	
81	organic substance catabolic process	2292	2.137E-09	1.698E-07	
82	multicellular organism development	7553	2.521E-09	1.979E-07	

83	positive regulation of blood vessel diameter	139	2.733E-09	2.119E-07	
84	regulation of cell-substrate junction organization	100	3.194E-09	2.448E-07	
85	detoxification	190	4.323E-09	3.273E-07	
86	enzyme linked receptor protein signaling pathway	1034	4.374E-09	3.274E-07	
87	developmental process	8666	4.560E-09	3.373E-07	
88	regulation of cell death	2629	4.671E-09	3.416E-07	
89	blood circulation	699	6.411E-09	4.636E-07	
90	response to nitrogen compound	2114	6.568E-09	4.697E-07	
91	blood vessel development	813	6.776E-09	4.792E-07	
92	cell junction organization	817	7.692E-09	5.381E-07	
93	negative regulation of phosphorylation	705	7.891E-09	5.461E-07	
94	regulation of apoptotic process	2371	8.787E-09	6.016E-07	
95	response to external stimulus	4027	9.345E-09	6.331E-07	
96	regulation of vasoconstriction	130	9.535E-09	6.393E-07	
97	regulation of cellular ketone metabolic process	282	1.044E-08	6.927E-07	
98	cell development	2582	1.114E-08	7.269E-07	
99	hormone metabolic process	312	1.118E-08	7.269E-07	
100	blood vessel morphogenesis	682	1.271E-08	8.181E-07	
