# Supplementary material for: Dietary excess regulates absorption and surface of gut epithelium through intestinal PPARα
Source: Nat Commun. 2021 Dec 2;12:7031. doi: 10.1038/s41467-021-27133-7 (PMC8639731; doi:10.1038/s41467-021-27133-7)
Supplement: Supplementary file 2 — Description of Additional Supplementary Files [file 41467_2021_27133_MOESM2_ESM.docx]

Description of Additional Supplementary Files

Title: Supplementary Data 1

Description: Metabolomic mass spectrometry source data
