# Supplementary material for: Dietary excess regulates absorption and surface of gut epithelium through intestinal PPARα
Source: Nat Commun. 2021 Dec 2;12:7031. doi: 10.1038/s41467-021-27133-7 (PMC8639731; doi:10.1038/s41467-021-27133-7)
Supplement: Supplementary file 4 — Reporting Summary [file 41467_2021_27133_MOESM4_ESM.pdf]

nature research

Corresponding author(s): Mirko Trajkovski

Last updated by author(s): Sep 10, 2021

Reporting Summary

Nature Research wishes to improve the reproducibility of the work that we publish. This form provides structure for consistency and transparency in reporting. For further information on Nature Research policies, see our Editorial Policies and the Editorial Policy Checklist.

Statistics

For all statistical analyses, confirm that the following items are present in the figure legend, table legend, main text, or Methods section.

n/a	Confirmed	
☐	☒	The exact sample size (*n*) for each experimental group/condition, given as a discrete number and unit of measurement
☐	☒	A statement on whether measurements were taken from distinct samples or whether the same sample was measured repeatedly
☐	☒	The statistical test(s) used AND whether they are one- or two-sided<br>*Only common tests should be described solely by name; describe more complex techniques in the Methods section.*
☐	☒	A description of all covariates tested
☐	☒	A description of any assumptions or corrections, such as tests of normality and adjustment for multiple comparisons
☐	☒	A full description of the statistical parameters including central tendency (e.g. means) or other basic estimates (e.g. regression coefficient) AND variation (e.g. standard deviation) or associated estimates of uncertainty (e.g. confidence intervals)
☐	☒	For null hypothesis testing, the test statistic (e.g. *F*, *t*, *r*) with confidence intervals, effect sizes, degrees of freedom and *P* value noted<br>*Give P values as exact values whenever suitable.*
☒	☐	For Bayesian analysis, information on the choice of priors and Markov chain Monte Carlo settings
☒	☐	For hierarchical and complex designs, identification of the appropriate level for tests and full reporting of outcomes
☐	☒	Estimates of effect sizes (e.g. Cohen's *d*, Pearson's *r*), indicating how they were calculated

Our web collection on statistics for biologists contains articles on many of the points above.

Software and code

Policy information about availability of computer code

Data collection	qRT-PCR was run on LightCycler 480 (Roche). H&E sections of proximal jejunum were scanned by Axioscan.Z1 platform (Zeiss) with 20x objective. Electron microscopy images were obtained using Morgagni microscope (FEI Company, Eindhoven, Netherlands) at EM Core Facility of Faculty of Medicine, University of Geneva. Crypt cultures were imaged by ImageXpress plate microscope. Western blot signal was detected with ECL reagent by GeneSys instrument. Bomb calorimetry was done in calorimeter Parr 6100. Lipoprotein in plasma was measured by cobas C111 platform. Other plasma measurements were read on SpectraMax Paradigm plate reader (Molecular Devices). Next Gen Sequencing of mRNA transcripts was performed on Ilumina HiSeq 2500 platform at iGE3 facility of the Institute of Genetics and Genomics of Geneva, University of Geneva.
Data analysis	Intestinal perimeters and villi were measured in ZEN 2 (Zeiss) and microvilli in iTEM (ResAlba). Lipid droplet quantification was from Oil Red O stainings, in ImageJ software by thresholding for lipid droplets, counting and measuring their area, then normalizing to total epithelial tissue area.<br>Statistical analysis was done in GraphPad Prism 8.<br>For RNASeq:<br>Sequencing: single read 100, TruSeq stranded, ribodepletion. The reads were mapped with the TopHat v.2 software to the UCSC mm10 reference; on new junctions and known junctions annotations. Biological quality control and summarization were done with PicardTools1.92.<br>The table of counts with the number of reads mapping to each gene feature of UCSC mm10 reference was prepared with HTSeq v0.6p1 (htseq-count). The differential expression analysis was performed with the statistical analysis R/Bioconductor package EdgeR v. 3.4.2, for the genes annotated in mm10. Briefly, the counts were normalized according to the library size and filtered. The genes having a count above 0.6 count per million reads (cpm), i.e. corresponding to 10 counts, in at least 3 samples were kept for the analysis. The differentially expressed genes tests were done with t-test, negative binomial distribution.<br>For pathway enrichment analysis, transcripts with log(count per million)>1 and p≤0.05 were selected and pathway analysis was done by MetaCore software pipeline (Thomson Reuters, portal.genego.com, 6.21.66768).<br>The cells were sorted on BD FACS ARIA Fusion, with software BD FACS Diva Software version 8.0.2. FACS plots have been produced by

FlowJoTM version 10.7.
Quantification of intestinal lipid droplets was done in FIJI ImageJ 1.51.

For manuscripts utilizing custom algorithms or software that are central to the research but not yet described in published literature, software must be made available to editors and reviewers. We strongly encourage code deposition in a community repository (e.g. GitHub). See the Nature Research guidelines for submitting code & software for further information.

Data

Policy information about availability of data

All manuscripts must include a data availability statement. This statement should provide the following information, where applicable:

- Accession codes, unique identifiers, or web links for publicly available datasets
- A list of figures that have associated raw data
- A description of any restrictions on data availability

Sequencing data associated with this study have been deposited to the Gene Expression Omnibus and are accessible by accession codes GSE74228, GSE182348, and GSE152056. Metabolomic source data are provided in the Supplementary Information of the paper. All figures have associated source data. Other data used in this study are available from the corresponding author upon reasonable request.

Field-specific reporting

Please select the one below that is the best fit for your research. If you are not sure, read the appropriate sections before making your selection.

[x] Life sciences [ ] Behavioural & social sciences [ ] Ecological, evolutionary & environmental sciences

For a reference copy of the document with all sections, see nature.com/documents/nr-reporting-summary-flat.pdf

Life sciences study design

All studies must disclose on these points even when the disclosure is negative.

Sample size	The number of animals in the experiments was calculated based on power calculation of 0.8, based on previous related experiments.
Data exclusions	No data has been excluded.
Replication	All animal experiments were repeated at least two times with similar results. RNASeq projects were performed once.
Randomization	The animals and samples were randomly assigned between the control and treatment groups.
Blinding	The animal experimentations were not blinded to the genotype and the treatment. Blinding was not possible because animals were readily identifiable by different food, temperature treatment, or genotyping marks, as well as practical reasons of colony maintenance and experiment planing.<br>For histological experiments, researchers were blinded to group allocation during processing and quantification of the sample.

Reporting for specific materials, systems and methods

We require information from authors about some types of materials, experimental systems and methods used in many studies. Here, indicate whether each material, system or method listed is relevant to your study. If you are not sure if a list item applies to your research, read the appropriate section before selecting a response.

Materials & experimental systems

n/a	Involved in the study	
[ ]	[x]	Antibodies
[x]	[ ]	Eukaryotic cell lines
[x]	[ ]	Palaeontology and archaeology
[ ]	[x]	Animals and other organisms
[x]	[ ]	Human research participants
[x]	[ ]	Clinical data
[x]	[ ]	Dual use research of concern

Methods

n/a	Involved in the study	
[x]	[ ]	ChIP-seq
[ ]	[x]	Flow cytometry
[x]	[ ]	MRI-based neuroimaging

Antibodies

Antibodies used	PLIN2 (guinea pig, 1:1000, MyBioSource, MBS534652), γ-tubulin (1:5000, Sigma Aldrich #T6557), GDH (rabitt, 1:1000, Cell Signalling, #12793,). GAPDH (rabbit, 1:1000, abcam, ab9485), PCNA (mouse, 1:500, origene, TA309795), PEPCK (1:1000, sc-74825, Santa Cruz).<br>For FACS: CD45-PE (eBioscience, 30-F11), CD31-PE (Biolegend, Mec13.3), Ter119-PE (Biolegend, Ter119), CD24-Pacific Blue (Biolegend, M1/69), and EPCAM-APC (eBioscience, G8.8), dilution of each 1:1000.

Validation	PLIN2: we validated it by the enrichment in the isolated lipid droplets and by negative tissue controls; also validated by others (DOI:10.1371/journal.pone.0131944; DOI: 10.1097/PAI.0000000000000004) and by the vendor.<br>γ-tubulin: numerous validations are available https://www.sigmaaldrich.com/catalog/product/sigma/t6557?lang=fr&region=CH<br>GDH and PEPCK: validated by WB of WT vs Glud1 or Pck1 I-KO intestinal lysate. Protocol using listed antibodies for FACS of Lgr5-GFP+ cells has been published many times, e.g. doi:10.1016/j.stem.2018.04.001 (2018), and we also confirmed identity of the sorted cell types by qPCR of cell-type markers.

Animals and other organisms

Policy information about studies involving animals; ARRIVE guidelines recommended for reporting animal research

Laboratory animals	Laboratory mice used:<br><br>C57BL/6J; ob/+; ob/ob;<br><br>Strains crossed with B6.Cg-Tg(Vil1-cre/ERT2)23Syr/J:<br>B6.129P2(Cg)-Hk2<tm1.1Uku<br>Pck1tm1.1Mgn/Mmnc<br>Glud1tm1.1Pma MGI:3835667<br><br>Strains crossed with B6·Cg-Tg(Vil1-cre)1000Gum/J:<br>Ppara loxP/loxP<br><br>Other strains:<br>Lgr5-EGFP-IRES-creERT2 (Jackson Laboratories, stock 008875)<br><br>Experimental mice were males, age 8-16 weeks old (unless specified otherwiise in the Figure legend).
Wild animals	The study did not involve wild animals.
Field-collected samples	Study did not involve samples collected from the field.
Ethics oversight	All procedures were approved by the animal care and animal experimentation authorities of the Canton of Geneva (licence GE/84/19).

Note that full information on the approval of the study protocol must also be provided in the manuscript.

Flow Cytometry

Plots

Confirm that:

- [x] The axis labels state the marker and fluorochrome used (e.g. CD4-FITC).
- [x] The axis scales are clearly visible. Include numbers along axes only for bottom left plot of group (a 'group' is an analysis of identical markers).
- [x] All plots are contour plots with outliers or pseudocolor plots.
- [x] A numerical value for number of cells or percentage (with statistics) is provided.

Methodology

Sample preparation	Isolated crypts from Lgr5-GFP mice were resuspended in 1ml of undiluted TrypLE Express (Invitrogen) + 100 µl of DNase I (10 U/µl, Roche), then incubated in a 32°C bath/thermobloc for 2min, without shaking, and placed on ice. Pelleted cells were washed once in Advanced DMEM/F12, and incubated 30 min on ice in antibody mix of 1µl of each CD45-PE (eBioscience, 30-F11), CD31-PE (Biolegend, Mec13.3), Ter119-PE (Biolegend, Ter119), CD24-Pacific Blue (Biolegend, M1/69), and EPCAM-APC (eBioscience, G8.8) in 1ml of Advanced DMEM/F12. Cells were washed twice in PBS and filtered through 70µm mesh. 7-aminoactinomycin D (7-AAD) was added to exclude dead cells.
Instrument	BD FACS ARIA Fusion.
Software	BD FACS Diva Software version 8.0.2. FACS plots have been produced by FlowJoTM version 10.7
Cell population abundance	Percentage of cell subtypes is displayed on FACS panel. Purity was checked by qPCR of stem cell (Olfm4), Paneth (Lyz1), progenitor and differentiated enterocyte gene marker (Alpi) in each sorted population.
Gating strategy	CD31− CD45− Ter119- 7-AAD+ were excluded, others isolated as follows: intestinal stem cells: Lgr5-EGFPhigh EpCAM+ CD24low/−; transiently amplifying progenitors: EGFPlow EpCAM+ CD24low/−; Paneth cells: CD24high/Sidescatterhigh EGFP− EpCAM+; unsorted epithelial cells from villus: EpCAM+.

- [x] Tick this box to confirm that a figure exemplifying the gating strategy is provided in the Supplementary Information.
